# Passively Targeted Curcumin-Loaded PEGylated PLGA Nanocapsules for Colon Cancer Therapy In Vivo

**DOI:** 10.1002/smll.201403799

**Published:** 2015-07-03

**Authors:** Rebecca Klippstein, Julie Tzu-Wen Wang, Riham I El-Gogary, Jie Bai, Falisa Mustafa, Noelia Rubio, Sukhvinder Bansal, Wafa T Al-Jamal, Khuloud T Al-Jamal

**Affiliations:** Institute of Pharmaceutical Science, King's College London, Franklin-Wilkins Building150 Stamford Street, London, SE1 9NH, UK E-mail: khuloud.al-jamal@kcl.ac.uk; Department of Pharmaceutics and Industrial, Pharmacy Faculty of Pharmacy, Ain Shams UniversityKhalifa El-Maamon Street, Abbasiya Square, Cairo, 11566, Egypt; School of Pharmacy, University of East AngliaNorwich Research Park, Norwich, NR4 7TJ, UK

**Keywords:** anticancer therapy, CT26 cells, hydrophobic-drugs, oil-core, single photon emission computed tomography/computed tomography (SPECT/CT), tumors

## Abstract

Clinical applications of curcumin for the treatment of cancer and other chronic diseases have been mainly hindered by its short biological half-life and poor water solubility. Nanotechnology-based drug delivery systems have the potential to enhance the efficacy of poorly soluble drugs for systemic delivery. This study proposes the use of poly(lactic-co-glycolic acid) (PLGA)-based polymeric oil-cored nanocapsules (NCs) for curcumin loading and delivery to colon cancer in mice after systemic injection. Formulations of different oil compositions are prepared and characterized for their curcumin loading, physico-chemical properties, and shelf-life stability. The results indicate that castor oil-cored PLGA-based NC achieves high drug loading efficiency (≈18% w(drug)/w(polymer)%) compared to previously reported NCs. Curcumin-loaded NCs internalize more efficiently in CT26 cells than the free drug, and exert therapeutic activity in vitro, leading to apoptosis and blocking the cell cycle. In addition, the formulated NC exhibits an extended blood circulation profile compared to the non-PEGylated NC, and accumulates in the subcutaneous CT26-tumors in mice, after systemic administration. The results are confirmed by optical and single photon emission computed tomography/computed tomography (SPECT/CT) imaging. In vivo growth delay studies are performed, and significantly smaller tumor volumes are achieved compared to empty NC injected animals. This study shows the great potential of the formulated NC for treating colon cancer.

## 1. Introduction

Nanotechnology-based drug delivery systems have the potential to enhance the efficacy of poorly soluble drugs for systemic delivery.[1] These features have made biodegradable polymeric nanocpasules (NCs) one of the most widely used. They can achieve high water insoluble drug loading, facilitate intratumoral distribution, and protect the active agent from premature degradation, allowing its sustained and controlled release.[2–4] NCs exhibit a typical core–shell structure in which active molecules are confined to an oil-core that is surrounded by a polymer membrane or coating.[5] Curcumin, has shown a wide range of pharmacological activities and has attracted great interest due to the possibility of tumor growth inhibition via multiple mechanisms.[6] Its anticancer potential is mediated through the inhibition and modulation of several intracellular signaling pathways leading to antitumor angiogenesis,[7] suppression of proliferation,[8] and prevention of metastasis[9,10] as confirmed in various in vitro and in vivo cancer studies. However, clinical applications of curcumin for the treatment of cancer and other chronic diseases have been hindered by its short biological half-life and poor aqueous solubility, resulting in poor bioavailability.[11] In vitro studies have established that curcumin-induced cancer cell death and promoted apoptosis at concentrations of 5 × 10^−6^–50 × 10^−6^ m and after several hours of exposure.[6] In another study, patients with advanced colorectal cancer were treated orally with curcumin at relatively high doses (3.6 g daily for four months); however, no partial responses to treatment were observed.[12] The development of an intravenous nanomedicine-based formulation of curcumin for the treatment of colon cancer is needed. Previous studies have shown that nanoparticles (NPs) have improved curcumin therapeutic effects when compared to free curcumin.[13,14] Few of these reports used PEGylated NPs and only one study reported curcumin formulation in PEGylated poly(lactic-co-glycolic acid) (PLGA)-lecithin NP for the treatment of colon cancer.[15] To our knowledge, no studies so far have investigated the therapeutic potential of curcumin NCs for the treatment of colon cancer in vivo after systemic administration.

In this work, oil core NC of high curcumin loading and optimum physico-chemical properties and shelf-life stability was prepared. The cellular uptake and cytotoxicity profile of PEGylated NCs were tested in CT26 cells in vitro. Organ biodistribution profile was studied by optical and single photon emission computed tomography/computed tomography (SPECT/CT) imaging. Tumor uptake and therapeutic efficacy were tested after multiple intravenous injections at 16 mg curcumin per kg dose in CT26 tumor bearing mice.

Overall, castor oil curcumin-loaded NC achieved ≈95% drug loading and exhibited sustained drug release profile in presence or absence of serum. Effective cell killing and apoptosis were achieved in CT26 cells in vitro. PEGylated NC showed good tumor accumulation and prolonged blood circulation profile facilitating enhanced permeation and retention (EPR) effect. In vivo therapeutic study suggests the promising potential of the reported delivery system for treating colon cancer using repetitive and high-dose therapy.

## 2. Results

### 2.1. Synthesis of PLGA Conjugates and Confirmation by FT-IR and ^1^H NMR

**Scheme 1:**
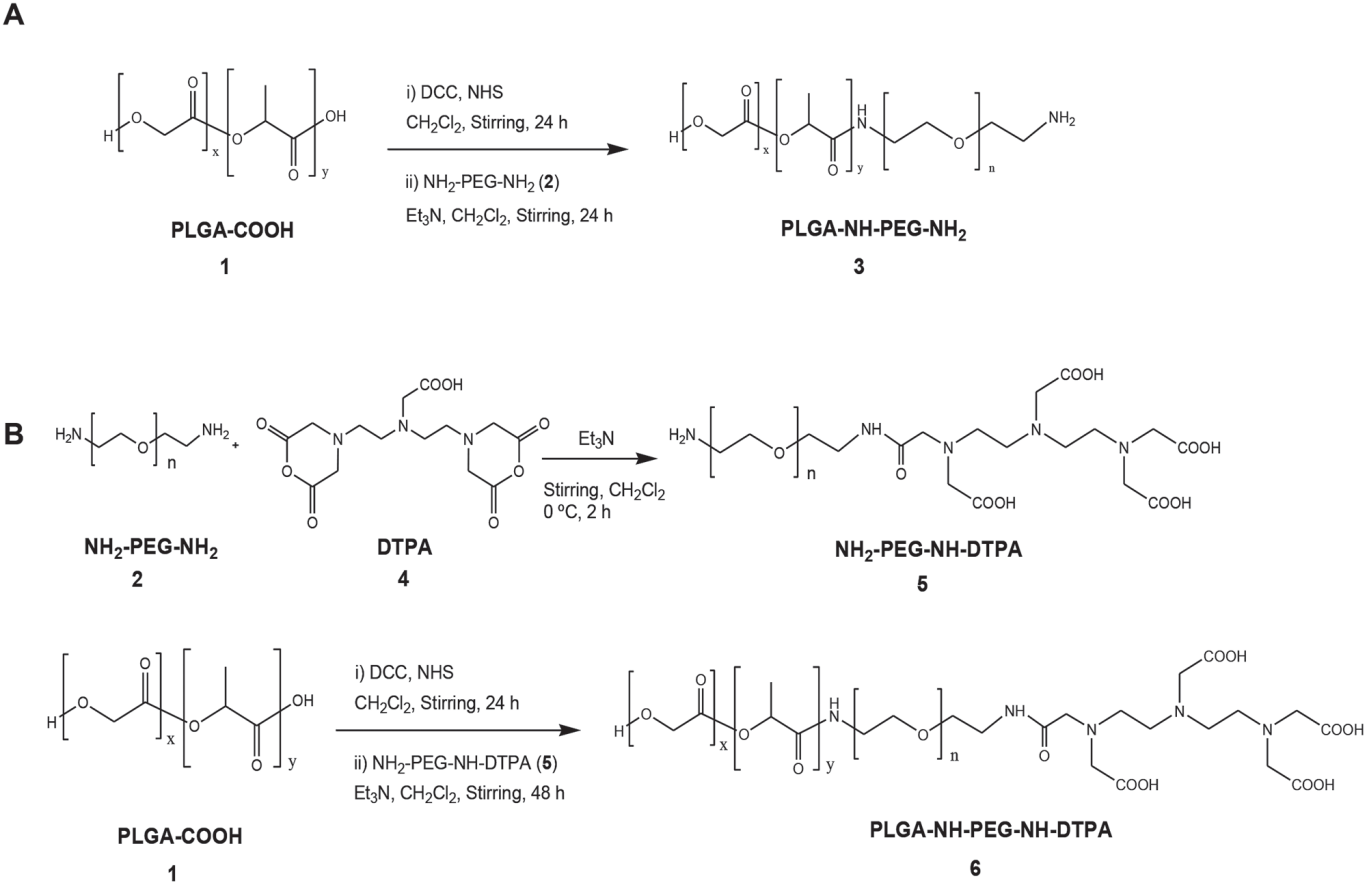
Synthesis of the conjugates. Conjugate 3 (PLGA_18KDa_-PEG_3.5KDa_-NH_2_) was used as a main polymer to formulate NC. Conjugate 6 (PLGA_18KDa_-PEG_3.5KDa_-NH-DTPA) contained the chelating molecule DTPA to facilitate radiolabeling of the conjugate with radioisotopes.

In this study, we aimed at synthesizing long-circulating PEGylated PLGA conjugates which are capable of encapsulating curcumin for subsequent cancer therapy in vivo. Moreover, the synthesis of radiolabeled conjugates was carried out in order to be able to track the NCs after systemic administration in tumor bearing mice. The synthetic approaches are schematically presented in **Scheme** **1**. This was based on reacting PLGA-COOH with excess PEG bis-amine to form PLGA–NH-PEG–NH_2_ conjugate (conjugate **3**). The excess of unreacted PEG bis-amine was removed by dialysis against deionized water while excess dicyclohexylcarbodiimide (DCC), *N*-hydroxysuccinimide (NHS), and dicyclohexylurea (DCU) were removed by precipitation of the polymer in diethylether where the by-products remain soluble. Characterization of PLGA–NH-PEG–NH_2_ (Conjugates **3**) was performed by FT-IR and ^1^H NMR (Figure S1, Supporting Information).

FT-IR chart (Figure S1C, inset, Supporting Information) confirmed the conjugation of PEG to the activated PLGA-COOH through the appearance of vibration frequencies at 1621 cm^−1^ (amide C=O) and 1563 cm^−1^ (N–H bond) that were absent in PLGA-COOH (compound **1**) and PEG bis-amine (compound **2**) charts (Figure S1A,B, Supporting Information). ^1^H NMR also confirmed the presence of PLGA and PEG protons on the NMR spectra. Peaks at 1.6, 4.8, and 5.2 ppm in ^1^H NMR were related to the CH_3_, CH, and CH_2_ protons of PLGA, respectively. The peak at 3.6 ppm accounted for the CH_2_ protons of PEG blocks (Figure S1C, Supporting Information). The presence of the free NH_2_ group in the synthesized polymer was confirmed by Ninhydrin/Keiser test (data not shown).

In order to be able to track the NC in vivo by gamma scintigraphy, PLGA-NH-PEG-NH-DTPA was synthesized to be incorporated into the NC during the formulation process. A two-step reaction was followed; PEG bis-amine was firstly reacted with DTPA (4:1). H_2_N-PEG-NH-DTPA (conjugate **5**) was purified by elution through SP-Sephadex C25 column. The presence of monofunctionalized H_2_N-PEG-NH-DTPA was confirmed by Ninhydrin test (data not shown).

**Figure 1:**
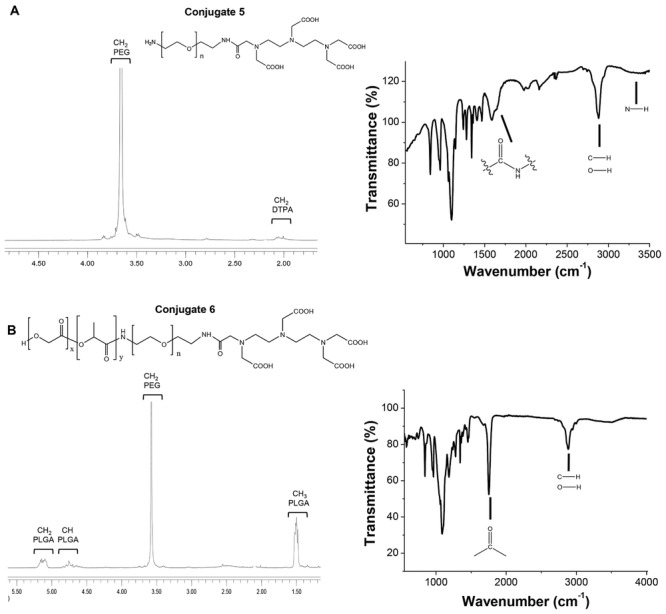
Characterization of the conjugates by ^1^H NMR and FT-IR. ^1^H NMR spectra (CDCl_3_) and FT-IR spectra (ATR mode) of A) conjugate 5 (NH_2_-PEG_3.5KDa_-NH-DTPA) and B) conjugates 6 (PLGA_18KDa_-NH-PEG_3.5KDa_-NH-DTPA).

Conjugation of DTPA to PEG bis-amine was confirmed by FT-IR (**Figure** **1**A) through the appearance of vibration frequencies at 1621 cm^−1^ (amide C=O) and 1563 cm^−1^ (N–H bond) and the reduction in the amine bond vibration at 3400 nm indicating coupling of one of the amine terminals. Conjugate **5** was then reacted with the activated form of PLGA-COOH resulting in the formation of PLGA-HN-PEG-NH-DTPA (conjugate **6**). Conjugate **6** was characterized by FT-IR and ^1^H NMR (Figure 1B). ^1^H NMR indicated the presence of PLGA protons (1.6, 4.8, and 5.2 ppm) in addition to PEG protons (3.6 ppm).

### 2.2. Quantification of PEG Contents in the Conjugates

For further characterization of the conjugates, the PEG content in conjugates **3** and **6** was measured with the BaCl_2_/I_2_ colorimetric assay as described in SI. PEG content was found to be in the range of 14.8%–16% of the total polymer weight. This indicated that 89.4%–92.5% of PLGA polymer was conjugated to PEG. This proves the efficiency of the applied methods in incorporating large amounts of PEG into the polymer.

### 2.3. Formulation and Physico-Chemical Characterization of NCs

**Scheme 2:**
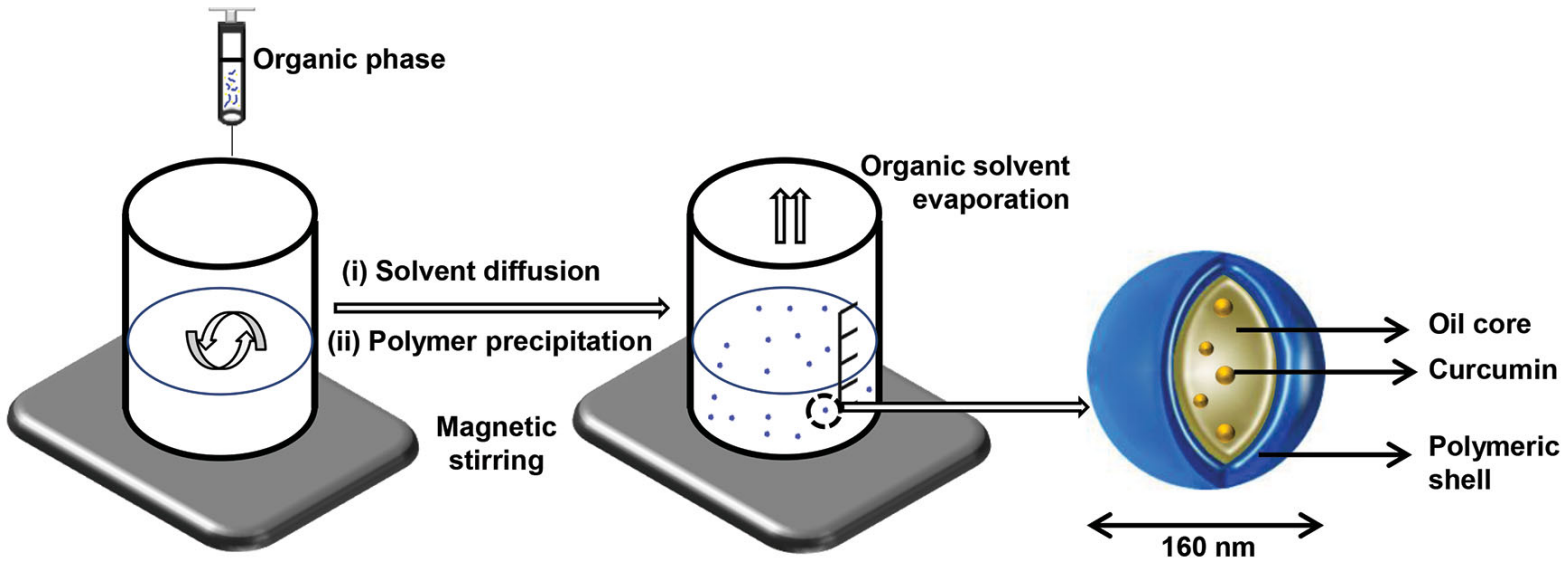
Preparation of curcumin-loaded NC by nanoprecipitation method. Schematic representation of the formulation method of the oil-cored NC. The organic phase composed of polymer, oil, lipophilic surfactant, and the drug dissolved in a water-miscible solvent is added dropwise to an aqueous phase containing a hydrophilic surfactant. Formation of NC takes place by solvent diffusion and polymer precipitation. Finally, the solvent is removed forming NC aqueous dispersion. The average diameter of NC is 160 nm.

After synthesis and confirmation of the structures of the conjugates, curcumin containing NCs were formulated using one of the following oils as a core material: (a) castor oil, (b) soybean oil or (c) miglyol812 oil. All formulations were prepared using the nanoprecipitation method (**Scheme** **2**). Unencapsulated drug was removed by gel filtration using water. The purified formulations were characterized for their hydrodynamic diameter, polydispersity index (PDI), and zeta potential immediately after purification and over 28 d storage at 4 °C (**Table** **1**). The hydrodynamic diameter ranged between 150 and 235 nm. On day 0, NC-castor oil exhibited significantly lower size (150.55 ± 4.73) compared to NC-soybean oil (235.42 ± 9.61) and NC-miglyol812 oil (206.5 ± 5.37). The PDI was ≤0.2 for the castor oil formulation but not for NC-soybean oil and NC-miglyol812 which showed a PDI value of 0.29 ± 0.01 and 0.26 ± 0.08, respectively, indicating greater heterogeneity of the sample.

**Table 1:** Shelf-life stability of oil-cored curcumin-loaded NCs prepared using different oil cores

	Day	Hydrodynamic diametera),b) [nm]	PDIa),b)	Zeta-potentialb),c) [mV]	Encapsulation efficiencyb),d) (EE%)	Loading efficiencyb),e) (LE%)
NC-castor oil	0	150.5 ± 4.7	0.16	−37.2	92.3 ±1.6	18.4 + 0.3
	7	140.1 ± 4.5	0.23	−38.2		
	14	156.2 ± 5.7	0.18	−37.3		
	28	142.5 ± 5.9	0.16	−38.6		
NC-soybean oil	0	235.4 ± 9.6	0.29	−45.3	68.9 + 2.9	13.7 + 0.6
	7	201.2 ± 1.3	0.23	−37.3		
	14	205.1 ± 8.6	031	−38.0		
	28	212.7 ± 2.8	0.29	−38.4		
NC-miglyol 812 oil	0	206.5 ± 5.3	0.26	−32.8	88 ±0.4	17.7 ± 0.1
	7	188.7 ± 7.0	0.29	−34.6		
	14	188.9 ± 11.4	0.17	−34.5		
	28	168.5 ± 3.2	0.16	−34.0		

a) Measured by dynamic light scattering;

b) xpressed as mean ± SD (*n* = 3);

c) Surface charge measured by electrophoresis;

d) Calculated as percentage of initial drug added, determined by spectrophotometry;

e) Calculated as mass of incorporated drug divided by the weight of polymer, determined by spectrophotometry.

NC-castor oil was found to be stable over the 28 d period storage with no significant changes in size, PDI or Zeta potential observed over time (Table 1). On the other hand, NC-soybean and NC-miglyol812 oil exhibited slight but significant increase and reduction in size, respectively, over the 28 d storage. No apparent change in color or phase separation was observed in any of the formulations.

The size and PDI of curcumin aggregates formed by their nano-precipitation method without PEGylated PLGA were 72.5 and 0.15, respectively. Micelles are expected to form due to solubilisation with lecithin and Tween 80.

### 2.4. Curcumin Loading and Encapsulation Efficiency

Initial screening for curcumin's solubility in oils used was carried out. Curcumin exhibited highest solubility in castor oil (1.91 mg mL^−1^) followed by miglyol812 (1.26 mg mL^−1^) and soybean oil (0.37 mg mL^−1^), at 37 °C. Drug encapsulation efficiency (EE%) and loading efficiency (LE%) for the different formulations were quantified by UV–vis spectroscopy and are described in Table 1. The highest and lowest EE% were achieved for NC-castor oil (≈92%) and NC-soybean oil (≈68%), respectively. Interestingly, EE% values were in agreement with the drug solubility values obtained for the drug in the different oils. Altogether NC-castor oil appeared to have the smallest size and PDI in addition to highest EE% and optimal shelf-life stability. It was therefore decided that further studies will be carried out using this formulation, thereafter referred to as NC. Drug-free NC is referred to as empty NC in this paper.

### 2.5. Drug Release Studies

**Figure 2:**
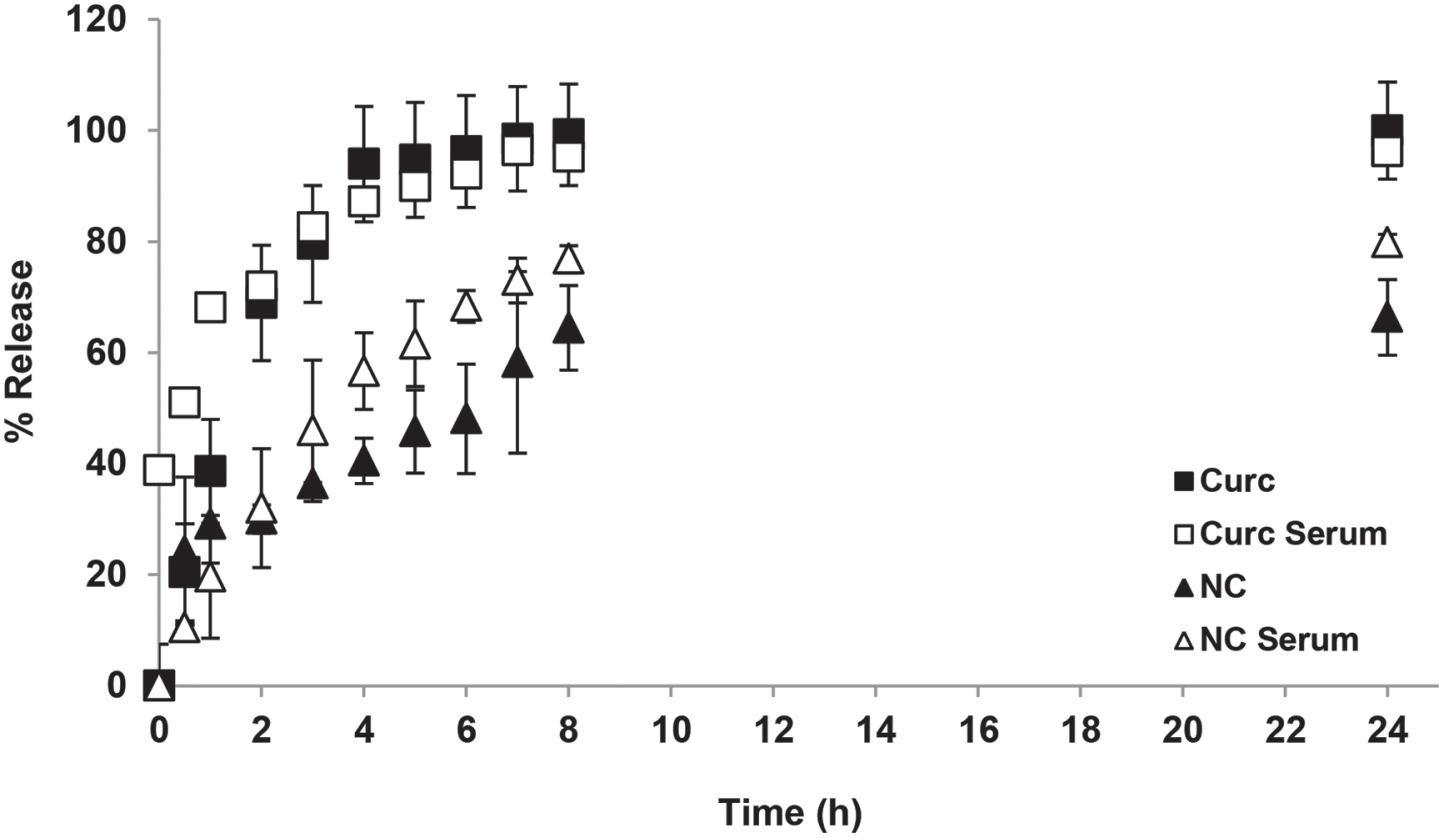
Release profile of curcumin from NC. NCs were dialyzed against 1% w/v Tween 80 in phosphate buffered saline (PBS), pH 7.4, in the presence and absence of 50% fetal calf serum. Drug concentration in the dialyzate was assessed by measuring the absorbance at 420 nm. A control experiment was set up concurrently in which the same amount of curcumin was dissolved in DMSO and dialyzed for comparison. NCs exhibited a sustained release pattern when compared to the free drug.

The release of curcumin from the PEGylated NCs was studied up to 24 h in the presence or absence of serum (**Figure** **2**). Curcumin dissolved in DMSO was dialyzed and used as the 100% release control. In case of curcumin/DMSO control, a burst effect was seen with 50% and 100% release achieved within the first 30 min and 4 h, respectively. Presence of serum did not affect its release profile. NC, on the other hand, showed a sustained drug release profile over the entire study period. The presence of serum significantly increased drug release at all time points. At 24 h, ≈66% and 80% of the drug was released from the NC in absence or presence of serum, respectively.

### 2.6. Intracellular Uptake of NC in CT26 Cells In Vitro

**Figure 3:**
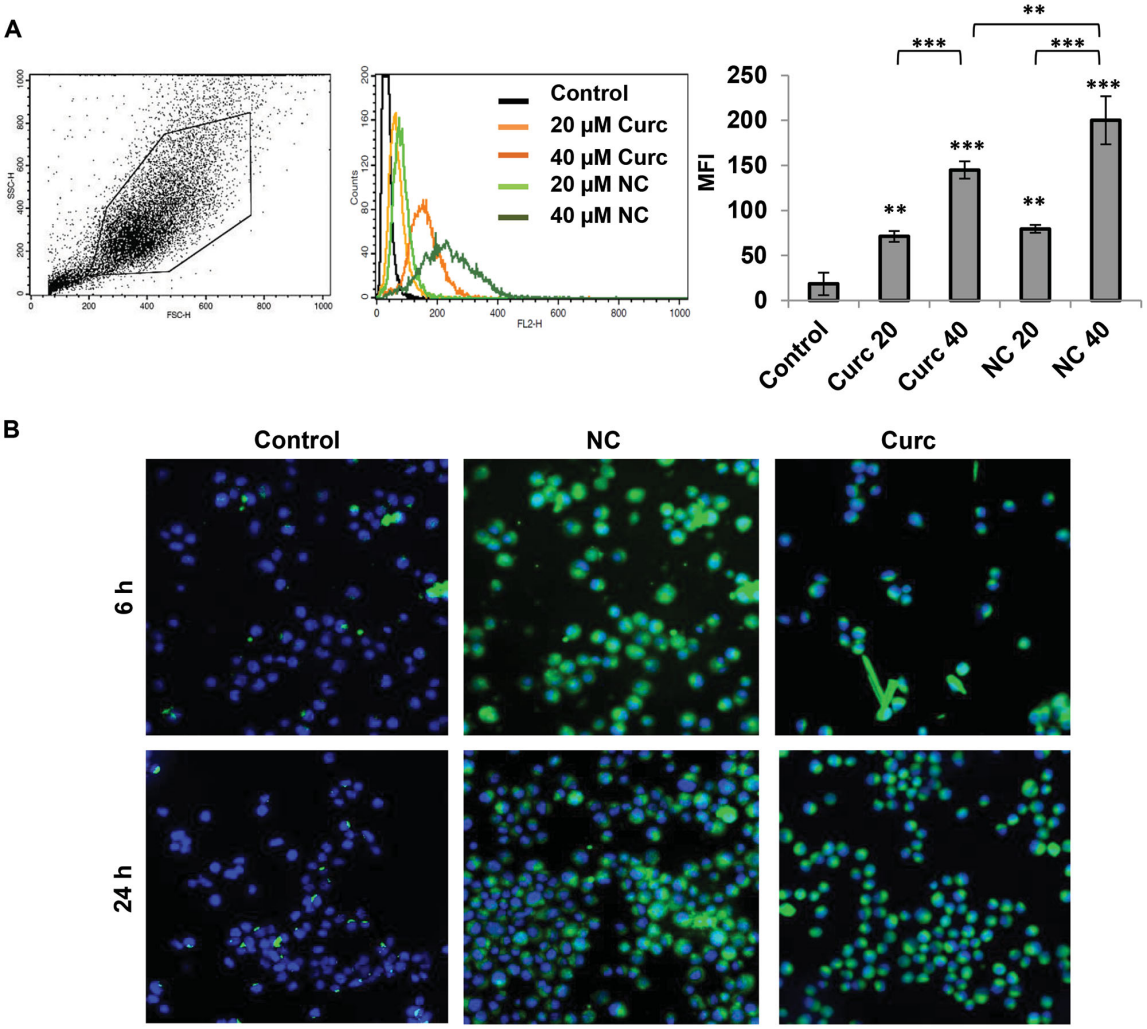
Intracellular uptake of NC in CT26 murine colon cancer cells in vitro. A) CT26 cells were incubated with either the free drug or the NC formulation for 6 h. Cellular uptake was assessed by measuring the mean fluorescence intensity (MFI) using flow cytometry and FL-2 detector. Drug uptake was enhanced, in a dose-dependent uptake pattern, when encapsulated in the NC. B) Cellular uptake was assessed by confocal laser scanning microscopy after 6 and 24 h. Curcumin uptake was indicated by green fluorescence inside the cells and nuclei were counterstained with DAPI. Values are expressed as mean ± SD (*n* = 3). *P < 0.05, **P < 0.01, ***P < 0.001 (one-way ANOVA test). Asterisks correspond to statistically significant differences between a particular group and control, unless indicated otherwise.

The intracellular uptake of intrinsically fluorescence curcumin in CT26 cells was expressed as mean fluorescence intensity (MFI) and measured with flow cytometry. CT26 cells were treated for 6 h with free curcumin or NC, at 20 × 10^−6^ m or 40 × 10^−6^ m. Cells treated with NC showed significantly higher MFI values compared to cells treated with free drug (**Figure** **3**A). The uptake was also concentration dependent on both treatments. MFI values of 79.9 ± 4.46 and 200.20 ± 26.7 were obtained for NC treatment at 20 × 10^−6^ and 40 × 10^−6^ m, respectively.

To confirm intracellular delivery of the NCs, CT26 cells were treated with free curcumin or NC (20 × 10^−6^ m) for 6 h and analyzed with confocal laser scanning microscopy (CLSM). Nuclei were counterstained with DAPI (blue) and uptake of curcumin was confirmed by the presence of green signals (Figure 3B). Results have confirmed the enhanced uptake of NCs in CT26 cells compared to free curcumin. Precipitates of the drug could sometimes be found in the case of the free drug. Microscopy and flow cytometry studies indicated effective drug uptake has been achieved with NCs.

### 2.7. Cytotoxicity and Apoptotic Activity in CT26 Cells In Vitro

**Figure 4:**
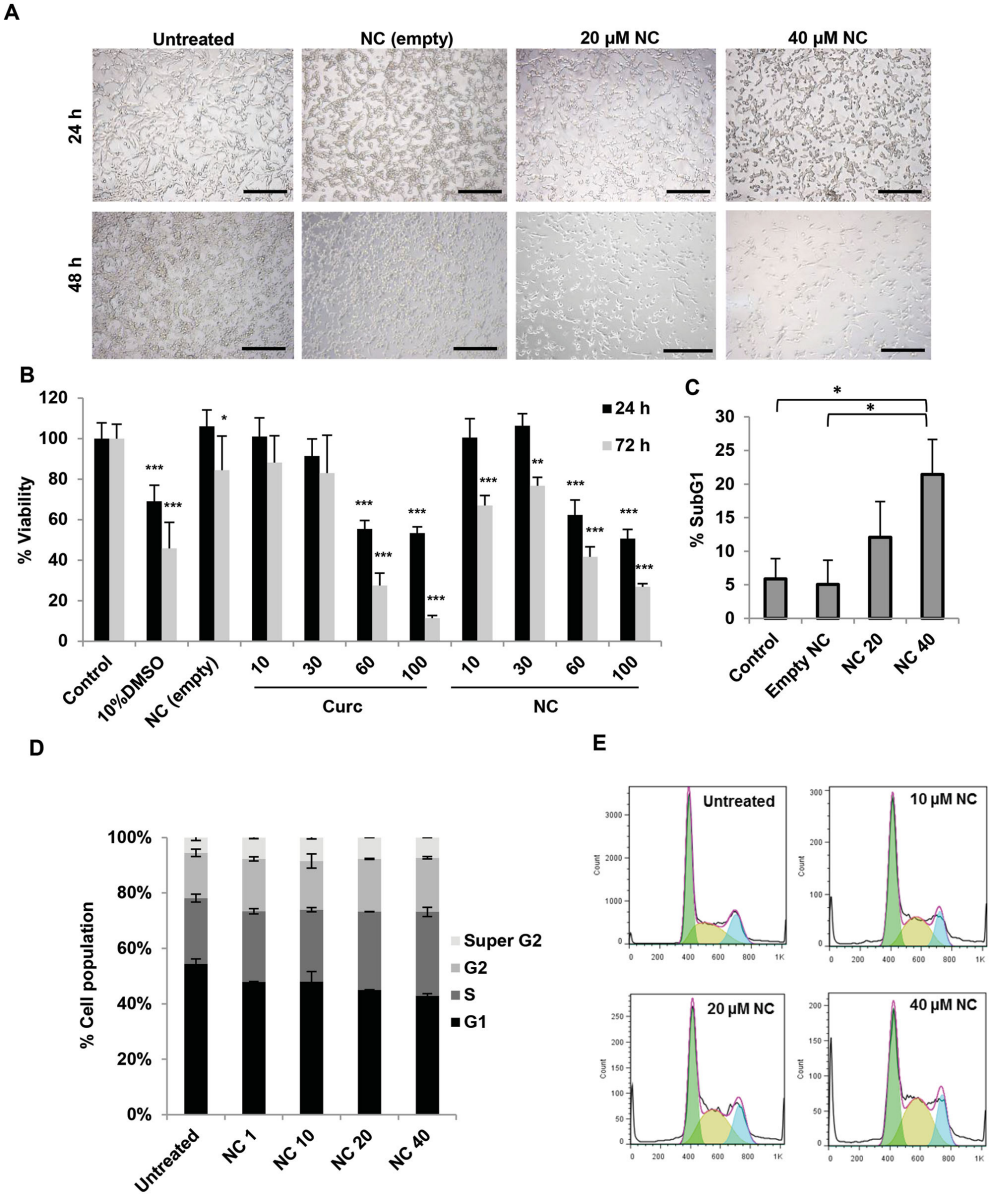
Cytotoxicity of NC in CT26 murine colon cancer cells in vitro and cell cycle distribution analysis. CT26 cells were incubated with NC for 24–72 h at increasing drug concentrations (from 10 × 10^−6^ to 100 × 10^−6^ m). A) Microscopic examination of CT26 cells after treatment with NC for 24 or 48 h. Cell rounding and reduction in cell number was observed for cells treated with drug-loaded NC in a time- and dose-dependent manner (scale bar 150 μm). B) Cell viability expressed as a percentage of control untreated cells, determined by MTT assay, was reduced as a function of drug concentration at both 24 and 72 h (*n* = 5). C) Percentage SubG1 cell population (apoptotic cells), determined using PI staining method, significantly increased, compared to untreated cells, when treated with 40 × 10^−6^ m for 48 h (*n* = 3). D) Relative changes in the percentage of cell cycle phases following 48 h of NC treatments. A shift in cell cycle distribution from G1 to S phase occurred in a dose-dependent manner (*n* = 3). Representative histograms showing the cell cycle distribution are shown in E). Values are expressed as mean ± SD. *P < 0.05, **P < 0.01, ***P < 0.001 versus control (one-way ANOVA test).

The cytotoxic effect of NC was initially screened in CT26 cells in vitro. Light microscopy images showed dose-dependent morphological changes when cells were treated with NC for 48 h, where cells appeared more round and smaller in size (**Figure** **4**A). As expected, cells treated with empty NC, at concentrations equivalent to that used in drug-loaded NC, showed normal cell morphology.

Cell viability was assessed with 3-(4,5-dimethylthiazol-2-yl)-2,5-diphenyltetrazolium bromide (MTT) assay at 10 × 10^−6^–100 × 10^−6^ m free drug or the NC. A dose-dependent reduction in cell viability was seen in both treatments, more significantly at 72 h compared to 24 h (Figure 4B). Interestingly, despite no significant differences in cell viability between the free drug and the NC at 60 × 10^−6^ and 100 × 10^−6^ m at both time points tested, NC showed more significant reduction in cell viability compared to the free drug at low concentrations (10 × 10^−6^ and 30 × 10^−6^ m) at 72 h.

Sub-G1 analysis (Figure 4C) allows quantification of hypodiploid cell percentage entering into the SubG1 phase. NC treatment resulted in a dose-dependent increase in the SubG1 cell population. Both assays therefore confirmed the anticancer effect of curcumin in CT26 cells.

### 2.8. Cell Cycle Distribution Analysis in CT26 Cells In Vitro

Cell cycle analysis was investigated at 24 and 48 h post-treatment (from 1 × 10^−6^ to 0 × 10^−6^ m NC) using flow cytometry. A shift in cell cycle distribution was detected at 48 h (Figure 4D,E) while no changes could be seen at 24 h (Figure S2, Supporting Information). NC treatment led to a steady increase in cell cycle arrest in S/G2 phase, upon increasing drug concentrations. Untreated cells and cells treated with 40 × 10^−6^ m NC showed ≈23.5% and ≈29.85% of cells in S/G2 phase, respectively. Additionally, untreated cells and cells treated with 40 × 10^−6^ m NC showed ≈16% and ≈19.3% of cells in G2 phase, respectively.

### 2.9. Tumor Uptake and Organ Biodistribution Studies In Vivo and Ex Vivo After Systemic Administration of NC

**Figure 5:**
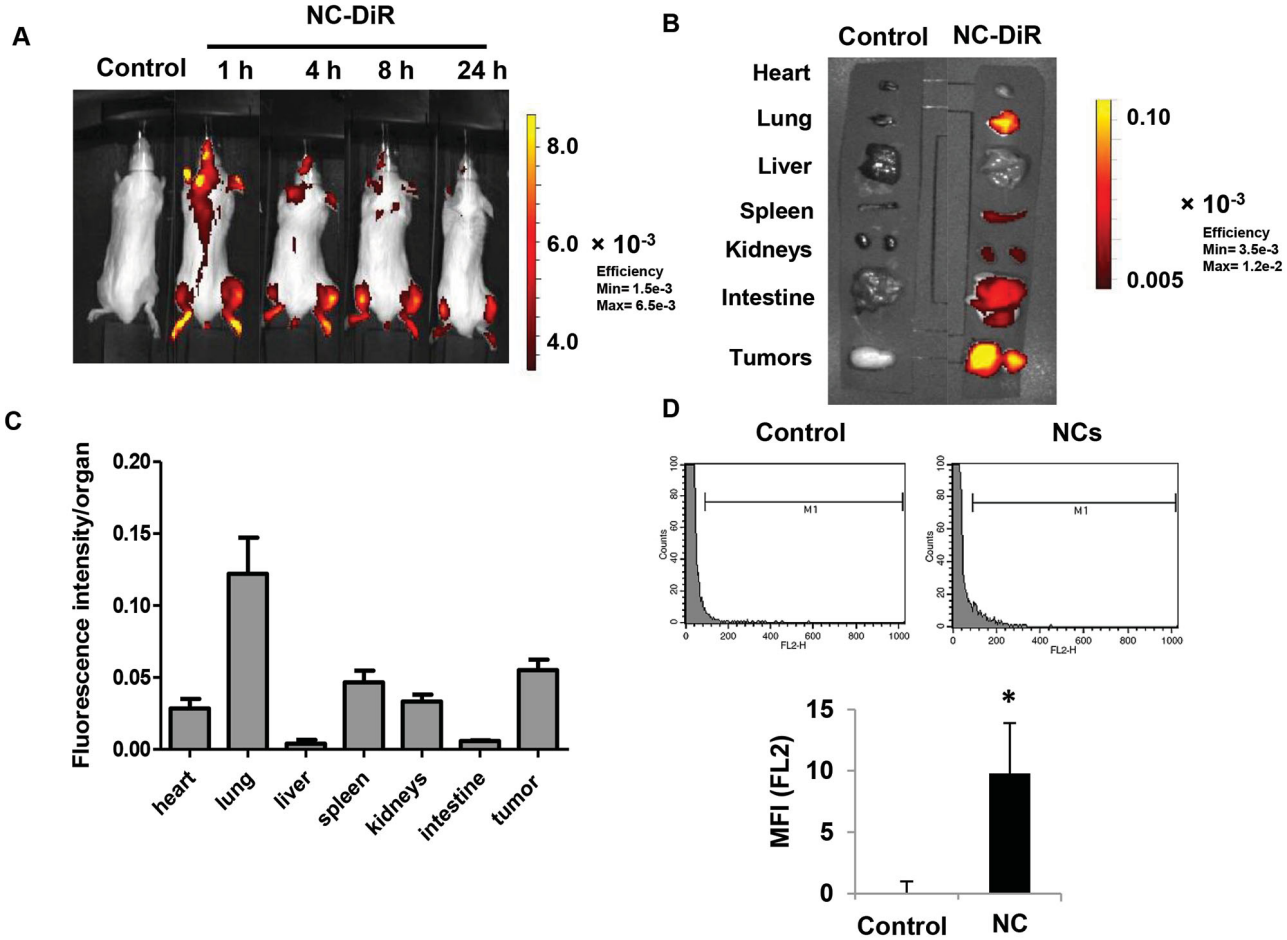
In vivo and ex vivo organ biodistribution of DiR-labeled NC in CT26 tumor bearing Balb/c mice after systemic administration. Mice were i.v. injected with PBS, NC-DiR or unlabeled NC. Dose injected was equivalent to 312.5 mg polymer per kg or 18.75 mg DiR per kg. A) Representative whole body in vivo images obtained at 1, 4, 8, and 24 h post-injection. B) Representative ex vivo images of excised organs at 24 h post-injection. C) Ex vivo quantification of fluorescence signals of DiR-labeled NCs per gram of excised organs and tumors at 24 h. All images were obtained by IVIS Lumina III at exposure time: 1 s; binning factor: 4; f number 2, field of view: D-12.5 cm; λex: 680, 700, 720, 740, and 760; λem: 790 nm. Data were analyzed by Living Image 4.3.1 Service Pack 2 software. For the purpose of quantification of curcumin in the tumor, mice were i.v. injected with curcumin-loaded non-DiR labeled NC (16 mg curcumin per kg). 24 h later, tumors were excised, homogenized, and uptake was quantified by flow cytometry. D) Representative histograms and mean fluorescence intensity (MFI). Values are presented as mean ± SD (*n* = 3), * *P* < 0.05 (Student's t-test).

In Vivo tumor accumulation of PEGylated NC was assessed using DiR-labeled NCs in colon cancer CT26 murine tumor models. Whole body imaging was carried out up to 24 h after injection followed by imaging of excised tissues. Tumor uptake of NCs was clearly observed in bi-focally implanted tumors when imaged from the dorsal view (**Figure** **5**A). On the other hand, no fluorescence signals were detected from naïve mice. The fluorescence image of excised organs is shown in Figure 5B where the highest fluorescence intensities were measured from the tumors compared to other organs. Fluorescence intensities from each organ were further quantified and the results are shown in Figure 5C. Taking into account the weights of the samples, slightly higher fluorescence signals per gram of tissue were calculated from lung than from tumors. Lower values were obtained from the rest of the tissues which was expected from the fluorescence image shown in Figure 5C.

To confirm curcumin uptake by tumor cells, non-DiR labeled curcumin-loaded NC was i.v. injected, tumors were excised 24 h later and tissue homogenate was assessed for drug fluorescence by flow cytometry (FL2) (Figure 5D). Higher MFI was observed for tumor homogenate of NC treated mice (84.2 ± 4.5) compared to saline injected mice (75.01 ± 1.01, * *P* < 0.05), indicating drug uptake by tumor cells.

### 2.10. Radiolabeling Optimization of DTPA-NC Formulations

For labeling of NCs, PLGA-NH-PEG-NH-DTPA (conjugate **6**) was synthesized and incorporated in NCs. DTPA-NCs were then chelated with ^111^In. The latter was selected due to its high capacity to be rapidly caged by DTPA with high thermodynamic equilibrium constant[16] in addition to its relatively long half-life (67.9 h), which allows imaging for up to 24 h. The radiolabeling efficiency (calculated as a percentage of ^111^In added to the labeling mixture) was tested using TLC technique after chelation ^111^In. Only [^111^In]-EDTA or [^111^In]-DTPA chelates migrated to the solvent front while the radiolabeled polymer remained at the application point.

**Figure 6:**
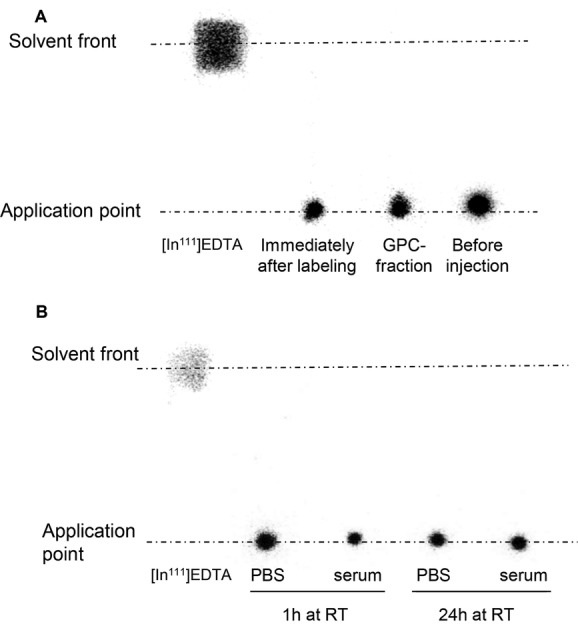
Radiolabeling of NC with ^111^In and radiolabel stability in serum. A) TLC of the radiolabeled NC immediately after radiolabeling, after desalting using PD10 desalting column and after concentrating the sample and just prior injection. No free ^111^In was detected in all the samples. B) TLC of radiolabeled NC after incubation with either mouse serum or PBS for 1 and 24 h at RT. No free ^111^In was detected indicating serum stability of the radiolabeled NCs.

In order to optimize the radiolabeling efficiency, NCs were prepared at increasing amounts of PLGA-NH-PEG-NH-DTPA (1% to 50% w/w) (Figure S3, Supporting Information). We concluded that inclusion of at least 5% of PLGA-NH-PEG-NH-DTPA in the NC formulation is needed to achieve 100% radiolabeling. Therefore, all the formulations prepared for subsequent studies were prepared at 5% w/w mass ratio. In order to exclude the possibility of nonspecific binding between ^111^InCl_3_ and the synthesized conjugate, NCs without DTPA were used as negative controls where no radiolabeling could be achieved thus confirming the chelation of ^111^In to DTPA molecules of the DTPA-NCs (Figure S3, Supporting Information). To eliminate the acetate buffer salts used during radiolabeling (pH 5.5), the NC suspension was desalted by passing it through PD10 size exclusion column and the NCs were resuspended in PBS prior injection to mice. The samples were concentrated to a final polymer concentration of 200 mg NCs per mL using the membrane centrifugation method. The absence of [^111^In]-EDTA in the injected material was confirmed once again after sample concentration and prior injection into the animals (**Figure** **6**A). No significant changes in hydrodynamic diameter or zeta potential were observed compared to non-DTPA NC (Table S1, Supporting Information).

### 2.11. Stability of NCs Radiolabeling in the Presence of Serum

Stability studies were assessed by incubating the radiolabeled NCs in either human serum (50% final concentration) or PBS at 37 °C for 24 h. The results shown in Figure 6B demonstrated high stability of all radiolabeled NCs in either human serum or PBS as all the ^111^In was still bound to the NCs, after 24 h incubation. It was demonstrated that the radiolabeled NCs were stable in serum so further in vivo studies were warranted.

### 2.12. SPECT/CT Imaging of Radiolabeled DTPA-NC In Vivo

**Figure 7:**
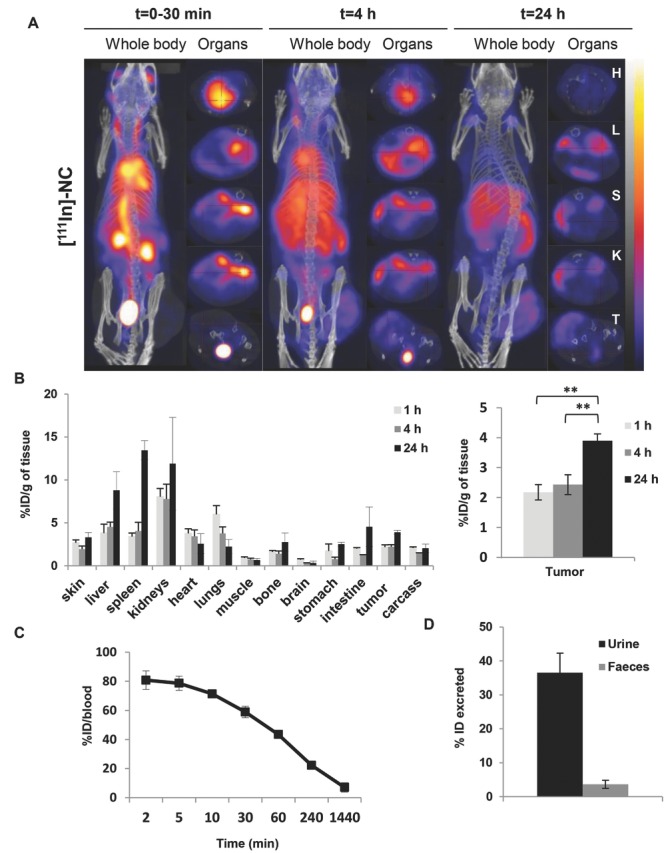
In vivo whole body 3D SPECT/CT imaging and biodistribution of NC-^111^In in CT26 tumor-bearing balb/c. Mice were i.v injected with NC-^111^In at a dose of 600 mg polymer per kg. Mice for SPECT/CT imaging with tumor inoculated at one side only while tumor inoculated bifocally for gamma scintigraphy studies. A) Whole body 3D SPECT/CT imaging at 0–30 min, 4 and 24 h post-injection with scanning time of 40–60 min each. Cross-sections were from heart (H), liver (L), spleen (S), kidney (KI), and tumor (T) at equivalent time points. Tumor accumulation was observed at 4 h post-injection and was enhanced over time. B) Organ biodistribution profile values were expressed as percentage injected dose per gram tissues (% ID/g) at 1, 4, and 24 h after injection of 600 mg PLGA-PEG/kg. Inset in A is showing the uptake in tumors. Blood clearance profile is shown in C); excretion profile at 24 h is shown in D). Signals were quantified by gamma scintigraphy. Results are expressed as means ± SD (*n* = 3–4). *P < 0.05, **P < 0.01 (one-way ANOVA test).

In Vivo organ biodistribution of radiolabeled PEGylated-NC is shown in **Figure** **7**A. The formulated NC exhibited extended blood circulation profile (Figure 7A,C) when compared to the non-PEGylated NC formulation, as the latter exhibited fast accumulation in the liver and spleen at 30 min (Figure S4, Supporting Information). Prolonged blood circulation profile of these NCs is shown by the brightness in the major blood vessels and the heart. Tumor accumulation becomes more intense at 4 h and lasts up to 24 h. Such results clearly confirmed fundamental differences in the organ biodistribution profile between the non-PEGylated and PEGylated NCs showing that the PEGylated NC reported in this study exhibit stealth characteristics, thus propose them as potential candidates to passively deliver drugs to the tumors. Overall, the results correlated well with optical imaging study (Figure 5).

### 2.13. Quantitative Biodistribution Study of Radiolabeled-NC in Mice after Systemic Administration

For quantitative determination of the NCs biodistribution and estimation of their uptake in CT26-tumor bearing Balb/c mice, radiolabeled NCs were injected via mouse tail vein at a dose of 600 mg (polymer) per kg. Mice were sacrificed at predetermined time intervals (30 min, 4 and 24 h) and the % injected dose (% ID)/gram tissue and % ID/organ were calculated and results were plotted in Figure 7B and Figure S5, Supporting Information. Blood clearance profiles were obtained and DTPA-NC showed 43 ± 2.99, 22 ± 2.09, and 7.7 ± 4.60% ID in the blood after 1, 4, and 24 h, respectively (Figure 7C). Significantly higher tumor uptake was achieved after 24 h (3.9 ± 0.22% ID/g) compared to 1 and 4 h. Highest % ID/g tissue was found in the liver, spleen and kidneys after 24 h. Almost 40% of the injected dose was found in the urine after 24 h (Figure 7D).

### 2.14. Quantitative Biodistribution Study of Curcumin in Mice after Systemic Administration

**Figure 8:**
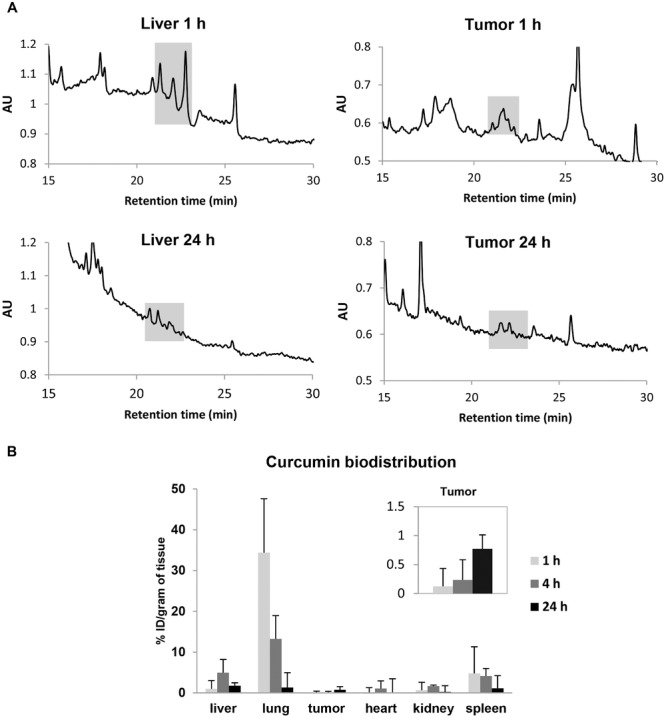
Organ biodistribution of curcumin in vivo by HPLC-FLd. Mice were i.v injected with curcumin-loaded NCs at a dose of 16 mg kg^−1^. A) Representative chromatograms of extracted curcumin from liver and tumor tissues after 1 and 24 h. Highlighted area shows curcumin peaks at retention times of 21.2, 21.8, and 22.5 min. B) Major organ biodistribution profile values for curcumin extracted from liver, lung, tumor (inset), heart, kidneys, and spleen at 1, 4, and 24 h post-injection, expressed as percentage injected dose per gram tissues (% ID/g). Curcumin uptake in tumors was observed at 1 h post-injection and was enhanced after 24 h. Results are expressed as means ± SD (*n* = 3).

Quantitative determination of curcumin biodistribution in CT26-tumor bearing Balb/c mice was performed using a high-performance liquid chromatography fluorescence detection (HPLC-FLd) analytical method. Mice were injected via tail vein at a dose of 16 mg curcumin per kg and sacrificed at predetermined time intervals (1, 4, and 24 h). The % ID/g tissue and % I.D/organ (**Figure** **8**B and Figure S6C, Supporting Information) were calculated using curcumin standard curves (Figure S6AB, Supporting Information). Commercially available curcumin consists of curcumin, demetoxycurcumin, and di-demethoxycurcumin.[17] These are observed at retention times of 21.2, 21.8, and 22.5 min and a single peak at 11.6 min was observed for dansyl-l-phenyl-alanine (internal standard).

The highest amount of curcumin was found in the liver, spleen, and lung after 1 h of injection. The % ID/g of tissue in these organs were 1.00% ± 2.02%, 4.80% ± 6.50%, and 34.37% ± 13.24%, respectively. Tumor accumulation was observed at 1 h (0.12% ± 0.31) of NC injection and was enhanced further at 24 h (0.77%. ± 0.24%) (Figure 8B, inset). Representative HPLC-FLd chromatograms are shown in Figure 8A. A reduction in curcumin peak area in the liver was observed overtime. In the case of tumor tissue, however, peak area appeared more pronounced after 24 h.

### 2.15. Tumor Growth Delay Studies in CT26 Tumor-Bearing Balb/c Mice

**Figure 9:**
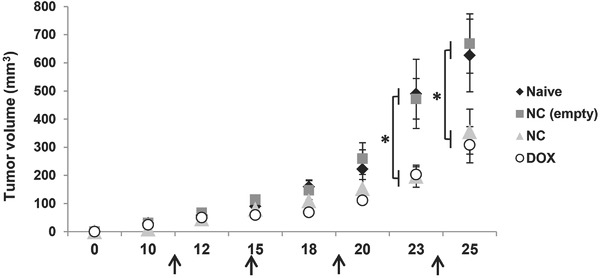
CT26 tumor growth delay in Balb/c after multiple injections of curcumin-loaded NC via tail vein. Mice were inoculated subcutaneously with 1 × 10^6^ CT26 cells. Animals were then randomly divided into four groups: Control (♦), empty NC (▪), curcumin-loaded NC (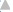), and DOX (positive control) (○). Mice were injected i.v. a total of 4 injections on day 11, 15, 19, and 24 post-tumor inoculation, at drug dose of 16 mg kg^−1^ curcumin (containing 250 mg polymer per kg) or 5 mg kg^−1^ DOX. Tumor size measurement was carried out three times a week. Data are given as mean value ± SEM (*n* = 10). Significant differences were examined using one-way ANOVA following by Tukey's multiple comparison test. *p < 0.05.

The anticancer potential of long circulating PEGylated curcumin-loaded NC was determined in CT26 mouse tumor bearing model. Mice were given four doses of curcumin-loaded NC (16 mg curcumin per kg) or the empty NC, on day 11, 15, 19, and 24. DOX (5 mg kg^−1^) was included as a clinically used anticancer agent. NC or DOX therapy resulted in significantly smaller tumor volumes compared to saline or empty NC injections, on day 23 and 25 (*p* < 0.05) (**Figure** **9**). On day 25, tumor volumes in saline, empty NC, DOX, and NC groups were 626 ± 71.98, 668 ± 122, 308 ± 64, and 355 ± 80, respectively. No significant differences between DOX and NC therapy were observed.

### 2.16. Organ Histological Examination and Terminal Deoxynucleotidyl Transferase dUTP Nick End Labeling (TUNEL) Assay on Tumor Tissue

**Figure 10:**
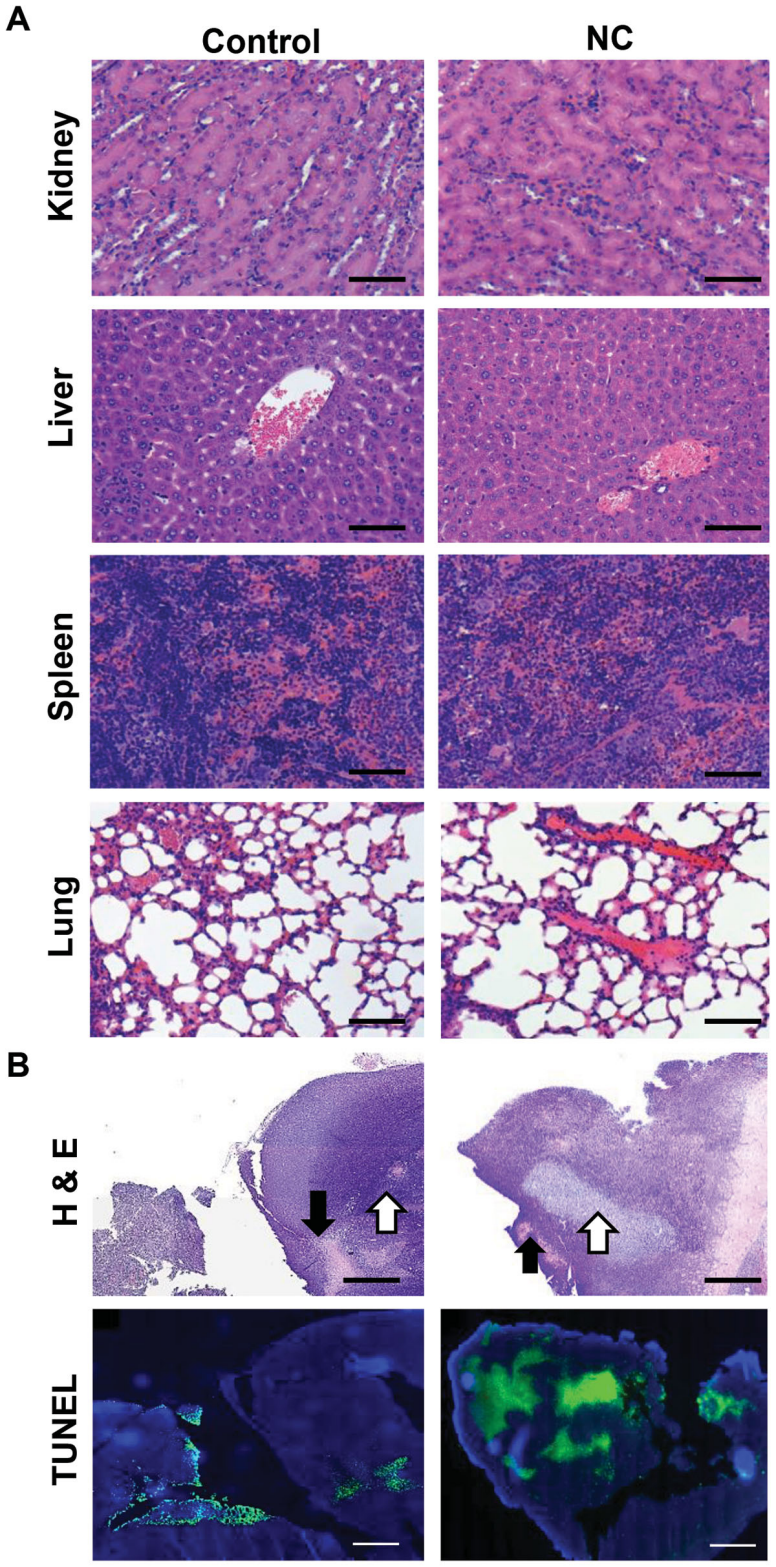
Histological examination of major organs and TUNEL staining of tumors after multiple administrations via tail vein. A) Haematoxylin and eosin (H&E) stained sections of kidney, liver, spleen, and lung in CT26 tumor-bearing Balb/c mice. Mice were injected i.v. a total of four injections on day 11, 15, 19, and 24 d post-tumor inoculation at drug dose of 16 mg curcumin per kg or with saline. No histological abnormalities were found in major organs. B) H&E and TUNEL stained tumor sections examined after multiple administrations of NC. Larger apoptotic (white arrows) and necrotic (black arrows) areas were detected in NC injected animals compared to control animals. TUNEL and DAPI were counterstained to identify apoptotic (green) cells from the total cell population (blue nuclei) in the bottom row. Scale bar 100 μm for H&E and 500 μm for TUNEL.

Histological analysis was carried out on H&E stained paraffin embedded tissue sections sampled after the treatment period (25 d). No significant histological changes were observed in lung, kidney, liver, and spleen compared to untreated tissues (**Figure** **10**A). Such results indicated the biocompatibility of the NC in vivo under the conditions tested. Apoptotic/necrotic areas were present in all tumors but larger apoptotic/necrotic areas were detected in NC treated tumors (Figure 10B). TUNEL assay was carried out to detect apoptotic cells. Apoptotic cells appear green, while other nuclei were counterstained using DAPI (in blue). As expected, large apoptotic areas were found in NC treated tumors, and were frequently found at the tumor mass periphery (Figure 10B).

## 3. Discussion

Several preclinical and clinical studies suggest that curcumin can supress cancer cell proliferation and promote cell death.[18–20] However, the major roadblocks in its clinical translation for cancer therapy are related to its poor water solubility, poor pharmacokinetics and limited bioavailability at the tumor site. In the current report, long-circulating curcumin-loaded oil-cored polymeric NCs were designed and formulated with the purpose of delivering high doses of curcumin to colon solid tumors, after systemic administration, for therapeutic applications in vivo. It was hypothesised that the inclusion of an oil core into the polymeric NC will offer superior drug loading compared to other nanoformulations, while polymer PEGylation can prolong the blood circulation profile, maximizing the enhanced permeation and retention in solid tumors so that the drug can reach the tumor at high concentrations. The biocompatibility of the constituting polymers allows for multiple injections for better therapeutic outcomes.

The novelty of the performed study lies in two aspects; the use of PLGA-based polymeric oil-cored nanocapsules for curcumin loading, and the application of this type of formulation for in vivo colon cancer therapy. In an attempt to formulate NCs of smallest size, narrow polydispersity index and highest drug loading, different oil core types were studied. It has been described in the literature that the oil viscosity and the oil surface tension are important parameters contributing to determining the NC and the drug entrapment.[21,22] Interestingly, in our study the oil core type did not only affect NCs size and PDI, but it also altered drug loading into the NC. The smallest hydrodynamic size and narrowest PDI were obtained with castor oil-cored NC (150 ± 4.73 nm). Castor oil is the most viscous and has the highest surface tension compared to soybean oil[23,24] and Miglyol812 oil.[21] The high drug loading obtained with castor oil-cored NC correlates well with the highest solubility of the curcumin in this oil.

It has been shown that curcumin targets multiple signaling pathways inducing cell cycle arrest[25] and that the principal mode of cell death induced by curcumin is apoptosis.[26] Several in vitro investigations have shown that curcumin inhibits cancer cell proliferation but high concentrations are needed.[27,28] In our study, MTT assay showed similar cell viability profile of curcumin-loaded NC to that of the free drug when tested in CT26 cells in vitro. This is in line with other curcumin nanoformulations, i.e., the cross-linked copolymer micellar aggregates,[29] and the self-assembled methoxy-poly(ethylene glycol) palmitate nanocarriers[30] where comparable cytotoxicity profiles were observed for the nanoformulations and the free curcumin, in non-colon cancer cell lines.

The in vitro therapeutic efficacy was not surprising as release studies showed that ≈60% and ≈80% of curcumin were released from the NC after 24 h, in presence of PBS and serum, respectively. Higher drug release in presence of serum is attributed to adsorption of serum proteins on NC and the subsequent destabilization effect, which has been reported with other curcumin NPs formulations.[15,31,32] Interestingly, cell imaging studies showed an improvement in curcumin uptake by CT26 cells when encapsulated in NCs compared to the free drug, as shown by flow cytometry and CLSM. The enhanced uptake is in agreement with other studies reporting curcumin nanoformulations such as EudragitS100 polymer,[33] monoolein lipids,[34] and chitosan particles,[35,36] designed for treatment of colon cancer in vitro.

Sub-G1 analysis method was used to quantify the percentage cell population undergoing late apoptosis or necrosis. A significant increase in the subG1 cell population was observed in cells treated with 40 × 10^−6^ m drug concentration (as NC). No adverse effects resulted from exposure to empty NC (no drug). Interestingly, similar findings were found by another group when testing free curcumin toxicity in colorectal cancer cells, using the same assay.[37] This was also in agreement with investigations utilizing the water-soluble albumin–curcumin conjugate, tested in noncolon cancer cell lines,[38] and curcumin NPs exposed to neuroblastoma,[39] glioblastoma,[40] and human squamous cell carcinoma in vitro.[41] When examining cell cycle progression, previous studies have shown that free curcumin inhibits the proliferation of cancer cell by blocking its G2/M phase.[42–44] The same effect was proven with curcumin PLGA nanoformulations tested in breast cancer[31,45] and leukaemia cancer cell lines.[46] Similarly, in our study, a concentration-dependent increase in cell population undergoing S/G2 phases, leading to a blockage into the G2/M phase was observed in CT26 cells.

Solid-cored polymeric nanoparticles including PLGA NPs,[13,31,47,48] amphiphilic methoxy poly(ethylene glycol)-polycaprolactone NPs,[49] cationic poly(butyl) cyanoacrylate NPs,[50] have been reported to encapsulate curcumin for cancer therapy. In an attempt to improve curcumin loading into the nanocarriers, other studies attempted the formulation of polymeric oil-cored nanocapsules made of polylactic acid (PLA) NCs[51] or poly(ε-caprolactone) (PCL) NCs.[52] To our knowledge this is the first study reporting formulation and optimization of PLGA oil-cored NCs for curcumin encapsulation.

Encouragingly, the formulated NC in this study presented higher curcumin loading than either the solid-cored PLGA NP[47] or the oil-cored NC made of non-PLGA polymers.[51,52] Previously reported solid PLGA NPs showed curcumin loading efficiency (w(drug)/w(polymer)%) ranging between 5%[15,31,53–56] and 15%.[57,58] Similar values were obtained for oil-cored polymeric NCs (≈5%–8%).[51,52] Interestingly, higher drug loading efficiency was achieved in our study (≈18%). Final solubility of curcumin in the NC aqueous dispersion obtained here was up to 1.4 mg mL^−1^. This is a great advantage as the same curcumin dose can be systemically injected, at a reduced polymer dose, compared to other studies.[15,54,59]

The second novel aspect of this study, directly linked to the aim of formulating polymeric NC of high drug loading, is performing the in vivo efficacy study in colon cancer model after i.v. injection. Higher drug loading is crucial as high curcumin dose is required to achieve a therapeutic effect in vivo.[60] Curcumin is a poorly water-soluble molecule (solubility in water is <0.1 mg mL^−1^)[61] which makes its systemic administration not possible.

There are dozens of formulations including liposomal curcumin,[62,63] PLA NCs,[51] PCL) NCs,[52] PLGA solid NPs,[13,32,47,64] amphiphilic methoxy poly(ethylene glycol)-polycaprolactone NPs,[49] cationic poly(butyl) cyanoacrylate NPs,[50,65] have confirmed the anticancer activity of curcumin nanoformulations in non-colon cancer cells in vitro[13,32,63,65] or in vivo.[47,49–52,62,64] To our knowledge, only two studies explored the efficacy of curcumin nanoformulations against colon cancer cells. The first study tested curcumin-loaded solid-cored PLGA NPs in vitro in HT29 cells,[15] while the second proved that curcumin-loaded monomethoxy poly(ethylene glycol)-poly(3-caprolactone) micelles can delay growth of C-26 colon cancer in vivo at a dose of 25 mg kg^−1^ after ten injections.[66] Neither of the two formulations was based on oil-cored polymeric NCs. In our study a dose of 16 mg kg^−1^ was administered four times to the mice.

A major advantage of this formulation is the improved shelf life stability[67] which is a problem encountered with nanoemulsions in spite of their high capacity to solubilize hydrophobic drugs.[68] The polymeric shell offers an additional protection mechanism for the therapeutic cargo, when compared to curcumin nanoemulsions where the polymeric shell is absent.[69] The stability provided by the polymeric shell and the ease of polymer's chemical modification, including PEGylation makes this system suitable for prolonged blood circulation profile thus maximising the enhanced permeation and retention in solid tumors.

The tumor growth delay observed when injecting the curcumin-loaded NC but not the non-medicated NC (Figure 9), is an indirect evidence of curcumin access to tumor cells.

To confirm the presence of curcumin in the tumor, a direct HPLC-FLd analytical method was used for curcumin quantification in major organs after intravenous administration of NC. Results showed that curcumin was distributed throughout the body with highest levels observed in the lung, spleen, and liver at 1 h. The highest curcumin levels in lung at 1 h can be explained by the fact that NC present in the blood stream, pass through the heart and circulate into the lungs. In this first stage of filtration, larger NC could have been trapped in small pulmonary capillary beds following intravenous injection of NCs.[70,71] Lung levels were reduced over time. These results are in accordance with radiolabeled NCs biodistribution studies, where a higher lung accumulation, compared to liver and spleen, was observed at 1 h. Highest level of the radiolabeled NCs was detected in the kidneys, probably due renal excretion of ^111^In-labeled polymer. Spleen and liver accumulation can be related to phagocytic cell uptake by the mononuclear phagocyte system.[72] Interestingly, the increase in radiolabeled-NCs levels in these organs over time was accompanied by a reduction in curcumin levels in these organs over time. This suggests that the drug is being metabolized into other forms or being excreted from the body. It is worth mentioning that the sensitivity of HPLC-FLd is limited compared to gamma counting so percentages detected by HPLC-FLd could be underestimated. As opposed to therapy experiments where multiple injections were performed, only a single injection was performed for HPLC-FLd analysis of organs. These results are in agreement with those described by Ravichandran[71] and Tsai et al.[73] where curcumin NPs showed preference to spleen and lung in rats, while the free curcumin showed liver and kidney accumulation, as determined by HPLC. Despite differences observed in the major organ biodistribution of curcumin and radiolabeled NCs in this study, tumor uptake of both the drug and the NC showed similar trends, with an increase in tumor accumulation from 1h to 24 h. Percentages of the drug in the tumor, like in other organs, were smaller than those obtained for the radiolabeled NCs presumably for the same reasons described above.

Additionally, qualitative flow cytometry studies on tumor tissue homogenates after 24 h of curcumin NCs is a direct evidence that curcumin reached tumor cells, indicating the ability of the drug to internalize into the cancer cells after extravasation. The fluorescence signals are not high due to the poor intrinsic fluorescence signals exhibited by curcumin (Figure 5D).[74] Finally, the hydrophobic dye (DiR) was encapsulated in the oil core of the NCs to mimic the hydrophobic curcumin. We observed by optical whole-body imaging that DiR reached the tumors (Figure 5) in a time-course, which correlates with SPECT/CT and biodistribution studies with gamma counting. This suggests that the DiR dye is stably encapsulated inside the NC. Altogether we hypothesise that the hydrophobic curcumin was stably encapsulated in the NC under the in vivo condition tested thus its biodistribution profile, at least the majority of it, can be matched to that of the NC.

Multiple injections (≈4 doses over 23 d treatment period) were carried out in this study and resulted in a significantly delayed tumor growth, comparable to that achieved with multiple injections of free doxorubicin. The dosage used for this study was 16 mg curcumin per kg which is in the range between 2.5 and 40 mg curcumin per kg used in the reports mentioned previously.[47,49,50,62,64] The therapeutic efficacy we observed with curcumin NCs is in line with other curcumin formulations or studies done on noncolon cancer models, which achieved a reduction between two[47,49,50,64] and three[62] times in the tumor volume. To our knowledge, this is the first attempt to deliver curcumin oil-cored NCs to colon solid tumors in vivo for therapeutic purposes.

## 4. Conclusion

This is the first report on formulating oil-cored PLGA-based polymeric NC encapsulating curcumin. The formulation was capable of (i) reaching CT26 solid tumors after i.v. injection, (ii) releasing the therapeutically active drug at tumor site; (iii) inducing apoptosis of proliferating tumor cells and (iv) delayed tumor growth in vivo while sparing healthy normal tissues. Herein, this data suggests that PEGylated NCs could be a promising curcumin carrier for colon cancer therapy but that high doses and repeated administrations may be the key to achieve an effective anticancer therapy.

## 5. Experimental Section

*Material and Reagents*: Dicyclohexylcarbodiimide (DCC), *N*-hydroxysuccinimide (NHS), anhydrous dimethylformamide (DMF), diethylenetriaminepentaacetic dianhydride (DTPA), 1,1′-dioctadecyl-3,3,3′,3′-tetramethylindotricarbocyanine iodide (DiR), 4′,6-diamidino-2-phenylindole (DAPI), dialysis tubing (MWCO 2000 Da), SP-Sephadex C-25 resin, and dansyl-l-phenylalanine were purchased from Sigma (UK). Polyoxyethylene-bis-amine (PEG-bis-amine, *M*_w_ ≈ 3500) was purchased from JENKEM USA. 75/25 DL-lactide/glycolide conjugate (PLGA-COOH, *M*_w_ ≈ 18000) was a gift from Purac Biomaterials. Curcumin was purchased from Santa Cruz Biotechnology (UK). Snake Skin dialysis tubing (MWCO 10 000 Da) was purchased from Thermo-fisher (USA). Soybean lecithin (Epikuron 140 V) was a kind gift from Cargill Pharmaceuticals. Medium-chain triglyceride (Miglyol812) was a generous gift from Cremer Oleo GmbH & Co. KG (GE). Castor oil, soybean oil, Tween 80, methylene chloride, acetone, absolute ethanol, dimethylsulphoxide (DMSO), and diethyl ether (ultra-pure grades) were obtained from Sigma-Aldrich (UK). RPMI-1640 media, fetal bovine serum (FBS), penicillin/streptomycin, trypsin/EDTA, and phosphate buffered saline (PBS) were obtained from Gibco, Invitrogen (UK). The radioactivity tracer [^111^In]Cl_3_ was obtained from Convidien UK commercial LTD (UK) as an aqueous solution and used without prior purification. Thin layer chromatography (TLC) strips for radiolabeling were purchased from Agilent Technologies UK Ltd (UK). DeadEnd Fluorometric TUNEL system was obtained from Pomega (UK). BD flow cytometry tubes were purchased from VWR (UK). Vectashield mounting media was from Vector Labs (UK); 16% formaldehyde, methanol-free, was from Thermo Scientific Pierce. Isoflurane (IsoFlo) for anaesthesia was purchased from Abbott Laboratories Ltd (UK). All reagents were used without further purification.

*Synthesis of the Conjugates*: Conjugate **3**. PLGA–NH-PEG–NH_2_ conjugate **3** was prepared according to the method developed by Yoo and Park, 2004 with some modifications.[18] Briefly, PLGA-COOH **1** (700 mg, 0.0412) together with a 4 molar excess of NHS (18.952 mg, 0.1648 mmol) and DCC (33.95 mg, 0.1648 mmol) were dissolved in anhydrous methylene chloride as presented in Scheme 1. The reaction mixture was stirred under nitrogen atmosphere for 24 h at RT for activation of carboxylic group of PLGA-COOH **1**. The resultant solution was filtered and added dropwise to a mixture of excess of PEG bis-amine **2** (721 mg, 0.206) and triethylamine (57.2 μL, 0.412 mmol) in anhydrous methylene chloride. PLGA-COOH: H_2_N-PEG-NH_2_ stoichiometric molar ratio was 1:5. The reaction mixture was stirred under nitrogen atmosphere for 24 h at room temperature (RT). The resultant solution was precipitated by the addition of ice-cold diethyl ether to remove dicyclohexylurea by-product, unreacted NHS and DCC. The precipitated product, PLGA–NH-PEG–NH_2_**3** was dried, dissolved in DMSO and dialyzed against deionized water for 2 d to remove excess unreacted PEG. Conjugate **3** was then lyophilized and stored at −20 °C till further use. PEG content was assessed colorimetrically upon reaction with barium chloride (in 1 N hydrochloric acid)/iodine solution as described in more details in the Supporting Information.

*^1^H NMR (400 MHz, CDCl_3_)*: *δ* = 5.05−5.2 (*m*, CH_2_ PLGA), 4.75–4.85 (*m,* CH PLGA), 3.5–3.6 (*m*, CH_2_ PEG), 1.42–1.6 (*m*, CH_3_ PLGA).

Conjugate **5**. PEG-bis amine **2** (347.4 mg, 0.102 mmol) and triethylamine (1.39 μL, 10.3 mmol) were dissolved in dichloromethane (20 mL). A solution of DTPA dianhydride **4** (9.11 mg, 0.026 mmol) was dissolved in dichloromethane (10 mL) and was added to the reaction mixture in a dropwise manner at 0 °C. After addition was complete, the solution was stirred for 2 h. After this period of time, the solvent was evaporated under reduced pressure and the solid obtained was dissolved in chloroform and precipitated in diethylether to remove unreacted DTPA. The obtained solid was subsequently dissolved in sodium acetate buffer (0.1 m, pH 4.6) and loaded onto a SP-Sephadex C25 cation-exchanger column and eluted using sodium acetate buffer affording DTPA-PEG-amine product (conjugate **5**). The product was monitored using Ninhydrin staining. The solution was dialyzed (MWCO 2000 Da) against deionized water and lyophilized. The as-obtained solid was dissolved in water and loaded onto a reverse phase C18 chromatographic column in order to remove free DTPA. The separation was carried out using a gradient of the solvents water and acetone. The as-obtained polymer was lyophililized affording conjugate **5** as a white solid (295 mg, yield: 79%). The compound was stored as powder at −20 °C till further use.

*^1^H NMR (400 MHz, CDCl_3_)*: *δ* = 7.6 (*bs*, NH_2_ PEG, 2H), 3.65 (*bs*, 320 H, CH_2_ PEG), 3.42 (*s*, CH_2_-COOH DTPA), 2.5–2.55 (*m*, CH_2_-N DTPA).

Conjugate **6**. PLGA-COOH **1** (268 mg, 0.015 mmol) was dissolved in 10 mL of dichloromethane together with a 4 molar excess of NHS (9.2 mg, 0.06 mmol) and DCC (17.60 mg, 0.06 mmol). The reaction mixture was stirred under nitrogen atmosphere for 24 h at room temperature. After this period of time, the solution was filtered and the solvent was evaporated under reduced pressure. The obtained solid was dissolved in chloroform and precipitated by the addition of ice-cold diethyl ether to remove dicyclohexylurea by-product, unreacted DCC and NHS, affording activated PLGA polymer as a white solid (270 mg, yield: 99%). The obtained compound (270 mg, 0.015 mmol) was dissolved in dichloromethane and added dropwise to a solution of conjugate **5** (295 mg, 0.075 mmol) and triethylamine (28 μL, 0.23 mmol) in dichloromethane. PLGA-COOH:H_2_N-PEG-NH-DTPA stoichiometric molar ratio used was 1:5. The reaction mixture was stirred under nitrogen atmosphere for 24 h at room temperature. After this period of time, the solvent was evaporated under reduced pressure and the product was dialyzed (MWCO 10 000 Da) against deionized water and lyophilized, affording conjugate **6** as a white solid (305 mg, yield: 93%). The compound was stored as powder at −20 °C till further use.

*^1^H NMR (400 MHz, CDCl_3_)*: *δ* = 5.0−5.2 (*m*, CH_2_ PLGA), 4.6–4.9 (*m*, CH PLGA), 3.65 (*bs*, 320 H, CH_2_ PEG), 1.4–1.6 (*m*, CH_3_ PLGA).

*Formulation of the NCs*: Curcumin polymeric NCs were prepared using the nanoprecipitation technique as described by Fessi et al.[75] and presented in Scheme 2. Briefly, polymer (25 mg), castor oil, Miglyol812 or soybean oil (300 μL), curcumin (5 mg), and soybean lecithin (25 mg) were dissolved in 5 mL of acetone/ethanol (60:40 v/v) mixture. This organic phase was added dropwise into the aqueous phase (10 mL) containing Tween 80 (0.2%) as a hydrophilic surfactant; the mixture was maintained under magnetic stirring in the chemical hood for 30 min to allow solvent to diffuse and form NCs. Organic solvents were then eliminated by evaporation under reduced pressure using a Buchi rotavap. The final volume of the colloidal suspension was adjusted to 10 mL.

*Solubility of Curcumin in Different Oils*: The solubility of curcumin in the different oils (castor oil, soybean oil, and miglyol812 oil) used for NC preparation was determined by simple saturation shake-flask method.[76] Briefly, excess amount of curcumin (4 mg) was placed in vials containing 1 mL of the oil, sealed and placed at 37 °C and shaken at 250 strokes min^−1^ for 48 h. Drug solubility was assessed by taking 100 μL of the supernatant and diluting in acetone and ethanol (ratio 3:2). The amount of curcumin dissolved in the organic solvent was determined using UV–vis spectrophotometer (Model UV-1601 PC; Shimadzu, Kyoto, Japan) by measuring the absorbance at 418 nm and the amount of curcumin able to solubilize in the oil was calculated from relevant standard plots.

*Determination of Encapsulation Efficiency (EE%)*: The encapsulation efficiency (EE%) of curcumin-loaded NCs was determined by measuring the amount of drug in the purified and unpurified nanocapsules suspension. In order to purify the sample and remove the nonencapsulated free drug, NCs were passed through a PD10 desalting column (GE Healthcare) and purified fractions were collected. To disrupt the NCs and quantify the total amount of drug in the purified and unpurified samples, 100 μL of the NCs preparations were diluted to 2 mL in ethanol and acetone (2:3 v/v). The amount of curcumin was determined using UV–vis spectrophotometer (Model UV-1601 PC; Shimadzu, Kyoto, Japan) by measuring the absorbance at 418 nm. A standard plot of known curcumin concentrations was prepared using the organic phase of the nanocapsules for each of the oil cores under identical conditions. The amount of curcumin in the measured samples was then calculated from the standard plots and the EE (%) was calculated using the following equation


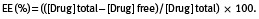


Drug loading efficiency (% LE) was calculated using the following equation


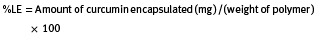


*Size and Zeta Potential Measurements*: Measurements of the average sizes and zeta potentials of the NCs were performed using dynamic light scattering (DLS) with a Nanosizer ZS Series (Malvern Instruments, Southborough, MA). Disposable polystyrene cells and disposable plain folded capillary Zeta cells were used. NC suspensions were diluted in deionized water and measurements were performed at 25 °C. Electrophoretic mobility was used to calculate the zeta-potential using the Helmholtz–Smoluchowski equation. The hydrodynamic size was presented as the average value for 20 runs, with triplicate measurements within each run.

*Release Profile and Serum Stability In Vitro*: Two millilitres of NC formulation containing 2.5 mg (curcumin) in 5 mL was taken and put into a 10 kDa dialysis bag. The dialysis membrane was dialyzed against 40 mL of 1% (w/v) Tween 80 in PBS, pH 7.4. Release was studied at 37 °C at 250 strokes min^−1^. At predetermined time points, 1.5 mL aliquots were taken and replaced by addition of an equal volume of the dialysate to maintain sink conditions. Drug concentration was assessed by measuring the absorbance at 420 nm using a Perkin–Elmer Lambda 35 UV–vis spectrophotometer, and calculated using a calibration curve for curcumin dissolved in the same media as the dialysate (*R*^2^ = 0.993) over the range between 1 and 10 μg mL^−1^). A control experiment was set up alongside in which the same amount of curcumin was dissolved in DMSO and dialyzed for comparison. This control was set up to eliminate nonspecific adsorption of the drug to the dialysis membrane.

*Shelf Life Stability*: NC suspensions were sealed in 7 mL glass vials and stored at 4 °C. The stability of NC dispersions were tested after 0, 7, 14, and 28 d of preparation by visual inspection of the physical properties (color and opacity) and also by size and zeta-potential measurements. The measurements were done in triplicate and presented as an average ± SD.

*Cell Culture*: The CT26 murine colon carcinoma (CT26; ATCC, CRL-2638) were cultured in Advanced RPMI media supplemented with 10% FBS, 50 U mL^−1^ penicillin, 50 μg mL^−1^ streptomycin, 1% l-glutamine, at 37 °C in 5% CO_._ Cells were routinely grown in 75 cm^2^ canted-neck tissue culture flasks and passaged twice a week using Trypsin/EDTA at 80% confluency.

*Uptake Studies In Vitro by Flow Cytometry*: Cells were seeded at a density of 5 × 10^4^ in 24-well plates, allowed to attach overnight and then treated with 20 × 10^−6^ and 40 × 10^−6^ m of curcumin (free drug in DMSO or as NC). After treatment, the cells were washed twice with PBS, trypsinized and centrifuged at 1500 rpm for 5 min and the cell pellet was resuspended in 250 μL of PBS. The internalization of curcumin or its NC was studied on 10 000 gated cells by detecting curcumin's fluorescence using FL2 channel detector and BD FACS Calibur flow cytometer (BD Biosciences). The measurements were done in triplicate and presented as mean ± SD.

*Uptake Studies In Vitro by Confocal Laser Scanning Microscopy*: CT26 cells were seeded onto glass coverslips at a density of 25 K cells per a well of a 24-well plates in RPMI media overnight. Cells were then incubated with 0.5 mL of 20 × 10^−6^ m NCs or free curcumin for 6 h. At the end of incubation period, cell were rinsed, fixed (200 μL of 4% PFA for 15 min at RT) and subsequently counterstained with DAPI. Coverslips were mounted on glass slides using VectaShield mounting media. Curcumin uptake was represented by the green signals, while nuclei were detected in blue. Confocal images were captured using a Nikon Eclipse Ti inverted microscope, Nikon, Tokyo, Japan, using an objective lens 20×/0.75NA 505 nm output filter, and a Plan-Neofluar 10 lens.

*Cytotoxicity Studies In Vitro*: CT26 cells were seeded in 96-well plates and incubated with different concentrations of curcumin or its NC (200 μL final volume) in complete media for 24 and 72 h. Cytotoxicity was examined by MTT assay. Briefly, at the end of the incubation period, media was removed and replaced with 120 μL of MTT solution at a final concentration of 0.5 mg mL^−1^. Cells were incubated for 3 h at 37 °C and 5% CO_2_. At the end of the incubation, formazan was dissolved in 200 μL of DMSO and the plate was read at 570 nm in a FLUO star OPTIMA plate reader (BMG Labtech) and the results were expressed as the percentage cell survival (mean ± SD) and calculated using the following equation: % Cell survival = (A570 nm of treated cells/A570 nm of untreated control cells) × 100.

*Assessment of Late Apoptosis and Necrosis by Sub-G1 Quantification*: Late apoptosis and necrosis induced by curcumin-loaded NC were measured by quantifying the fraction of cells displaying reduced amount of DNA, named sub-G1 population. CT26 cells were seeded at 5 × 10^4^ cells per well in 24-well plates and incubated overnight at 37 °C with 5% CO_2_. Cells were then treated with 20 × 10^−6^ and 40 × 10^−6^ m of NC. At the end of treatment period, floating cells were collected and adherent cells were trypsinized and transferred into BD flow cytometer tubes. After centrifugation at 800 × g for 5 min and washing in PBS, cells were resuspended in 100 μL of PBS and 900 μL of 70% cold ethanol for fixation and stored overnight at 4 °C. Fixed cells were then centrifuged, washed in PBS, and treated in a mix of 250 μL of PBS and 250 μL of DNA extraction buffer (Na_2_HPO_4_ 190 × 10^−3^ m, Citric Acid 4 × 10^−3^ m, pH 7.8) for 10 min at 37 °C. Samples were then centrifuged and resuspended in 50 μL of RNase in PBS (100 μg mL^−1^) before being stained for 30 min at 37 °C in the dark with 400 μL of propidium iodide (PI) solution (40 μg mL^−1^ of PI in PBS). Propidium iodide fluorescence was analyzed by flow cytometry using a BD FACS Calibur flow cytometer obtained from BD Bioscience (US). Finally, 10 000 cells were gated and fluorescence of Sub-G1 population was analyzed in triplicates for each condition using the FL2 detector. Results were expressed as average ± SD (*n* = 3).

*Cell Cycle Analysis by Flow Cytometry*: To determine the percentage of cells that exist in specific phases of the cell cycle, flow cytometry analysis was performed. Briefly, CT26 cells were seeded at a density of 5 × 10^4^ in 24-well plates, allowed to attach overnight and then treated with 1 × 10^−6^, 10 × 10^−6^, 20 × 10^−6^, and 40 × 10^−6^ m of NCs. Upon treatment, cells were washed with PBS, trypsinized and transferred into BD flow cytometer tubes. After centrifugation at 800 ×*g* for 5 min and washing in PBS, cells were resuspended in 100 μL of PBS and 900 μL of 70% cold ethanol for fixation for 1 h. Fixed cells were then centrifuged, washed in PBS, and treated with 50 μL of RNase in PBS (100 μg mL^−1^) before being stained for 30 min at 37 °C in the dark with 400 μL of propidium iodide (PI) solution (40 μg mL^−1^ of PI in PBS). Propidium iodide fluorescence was analyzed by flow cytometry using a BD FACS Calibur flow cytometer obtained from BD Bioscience (US). Finally, 10 000 cells were gated and fluorescence was analyzed in triplicates for each condition using the FL2 detector and FlowJo software. Results were expressed as averages of percentage cell populations in each phase of the cell cycle ± SD (*n* = 3).

*Organ Biodistribution Studies In Vivo by Optical Imaging*: All animal experiments were performed in compliance with the UK Home Office (1989) Code of Practice for the housing and care of Animals used in Scientific Procedures. Female Balb/c mice (≈20 g) aged-4–6 weeks (Harlan) were inoculated subcutaneously with CT26 cells (1 × 10^6^ cells in 0.1 mL PBS) at both lower flanks. Mice (*n* = 3) were intravenously injected with prepared DiR-labeled NCs in PBS solution at a dose of 312.5 mg PLGA_18KDa_-PEG_3.5KDa_-NH_2_/kg and 18.75 mg kg^−1^ DiR.and scanned at 1, 4, and 24 h post-injection using an IVIS Lumina Series III In Vivo Imaging System (Caliper Life Sciences, Perkin Elmer, USA). Untreated animals were also included as controls. Animals were anesthetized with 1.5% isoflurane/98.5% oxygen to maintain sedation during the imaging procedure. Ex vivo imaging was carried out immediately afterward by imaging excised major organs (heart, lung, liver, spleen, and kidney) including tumors. Fluorescence images was obtained using DiR filter (680, 700, 720, 740, and 760/790 nm for excitation/emission wavelengths) with exposure time of 1 s. All images were captured using a sequential acquisition spectra unmixing mode and were obtained using binning factor of 4, f number of 2, and field of view of D-12.5 cm. The collected fluorescence emission signals were stored in efficiency units.

*Radiolabeling of DTPA-Conjugates*: In order to carry out the biodistribution studies, NCs were formulated using non-DTPA containing copolymers (conjugate **3**) with increasing amount of conjugate **6** (PLGA-HN-PEG-NH-DTPA). Conjugate **6** was incorporated at 1%–50% in the formulation. NCs were then radiolabeled with Indium (^111^In). ^111^In was selected as the radioisotope for its capacity to rapidly be caged by DTPA with high thermodynamic equilibrium constant.[16]^111^In decays by electron capture with a physical half-life of 67.9 h, and others have previously shown that [^111^In]DTPA complex exhibits a strong chelating effect in the presence of human serum.[77] The labeling with ^111^InCl_3_ was performed according to the method described before.[78] NCs were mixed (1:1(v/v)) with 0.2 m ammonium acetate buffer (pH 5.5) containing ^111^InCl_3_ for 30 min at room temperature. According to the sensitivity of each study, the quantity of radioactivity was adjusted. After 30 min, the reaction was stopped by adding of 0.1 m EDTA solution to the mixture, to chelate the free ^111^In. Unchelated ^111^In was then removed using PD-10 desalting columns (GE Healthcare, UK) and the pH of the solution was adjusted to pH 7.0–7.4 at the same time due to the use of PBS as the equilibration buffer. Solutions were later concentrated to the required volume for injection by centrifugal filtration (Amicon Ultra-4, Merck Millipore, UK) at 4000 × g.

*Efficiency and Stability of the Radiolabeling*: Aliquots of the radioconjugates or [^111^In]EDTA were diluted with PBS [1:5 (v/v)] and then spotted in glass microfiber chromatography paper impregnated with silica gel. The strips were allowed to dry and developed with mobile phase of 50 × 10^−3^ m EDTA in 0.1 m ammonium acetate. Strips were placed on a multipurpose storage phosphor screen (Cyclone, Packard, Japan) and kept in an autoradiography cassette (Kodak Biomax Cassette) for 15 min. Then, quantitative autoradiography counting was performed using a cyclone phosphor detector (Packard, Australia). The labeling stability was tested by incubation of the radioconjugates in the presence or absence of CD1 mouse serum. Aliquots were diluted in 50% mouse serum or PBS [1:5 (v/v)], and incubated for 0 min, 60 min and 24 h at RT. The percentage of [^111^In]NC (immobile spot) or [^111^In]EDTA (mobile spot) was evaluated by TLC, using the same protocol described above.

*Tissue Biodistribution by Gamma Scintigraphy*: CT26 tumor bearing Balb/c mice (*n* = 3–4 per time point) were injected via the tail vein (i.v.) with 250 μL of the NCs dispersion at a dose of 600 mg kg^−1^ polymer-[^111^In]-labeled NC and 0.2–0.5 MBq of radioactivity. At the end of each time-point, the animals were sacrificed and the major organs (brain, lung, liver, spleen, kidney, heart, stomach, intestine, muscle, skin, and bone (Femur)) were collected, weighted, and placed in preweighted scintillation vials. Each organ was analyzed for [^111^In] specific activity using an automated gamma counter (LKB Wallac 1282 Compugamma, PerkinElmer, UK) together with dilutions of injected dose with dead time limit below 60%. The gamma rays emitted by the radioisotope were detected, quantified and corrected for physical radioisotope decay by the gamma counter. Radioactivity readings (counts per minute – CPM) were plotted as percentage of injected dose per organ or percentage of injected dose per gram of tissue. The data were expressed as the mean of triplicate or quadruplicate samples ± SD.

*Tumor Uptake by Live Animal SPECT/CT Imaging in Tumor-Bearing Mice*: Anaesthetized CT26 tumor bearing Balb/c mice (*n* = 2) were injected intravenously with 250 μL of 600 mg kg^−1^ polymer-[^111^In]-labeled NC and 10–12 MBq of radioactivity. Mice were imaged with nanoSPECT/CT scanner (Bioscan, USA) at different time points: immediately after the i.v. administration (0–30 min), after 3.5 and 24 h. For each mouse, a tomography was initially done (45 Kvp; 1000 ms) which allowed the calculation of certain parameters used in the SPECT and CT scanner, such as the starting line, finish line and axis of rotation of the acquisition, characteristics of each mice. SPECT scans were obtained using a 4-head scanner with 1.4 mm pinhole collimators and the following settings: number of projections: 24; time per projection: 60 s; duration of the scan 60 min. CT scans were obtained at the end of each SPECT acquisition using 45 Kvp. All data were reconstructed with MEDISO (medical Imaging System) and the fusion of SPECT and CT acquisitions were carried out using PMOD software.

*Curcumin Extraction from Tissue Samples and Analysis by HPLC-FLd*: The extraction solvent was prepared from 98% acetonitrile and 2% TFA (v/v). For method validation, curcumin-loaded NCs were added to liver tissue at different concentrations from 0.25 to 8.0 μg mL^−1^. Spiked liver was transferred into 15 mL test tubes and vortex-mixed for 20 s. 1 mL of 1X PBS was added and samples were homogenized using an ULTRA-TURRAX T18 Homogenizer (IKA, GE). After the tissue was completely homogenized, 2 mL of the extraction solvent was added and were left under agitation overnight. Samples were then centrifuged at 3000×*g* for 5 min at 4 °C and 1 mL of the supernatant was transferred to a clean glass tube. The extraction was repeated twice with 1 mL of extraction solvent, and the pooled supernatants were concentrated using a centrifugal evaporator-Genevac EZ2 (Genevac Ltd, UK) at low heat setting (40 °C). The content was transferred to an injection sample vial, and 50 μL was injected into the HPLC-FLd system (Agilent Technologies) using a Phenomenex C18 column (150 × 2.1 mm, 3.6 μm) and a flow rate of 0.2 mL min. The mobile phase consisted of A: 0.1% TFA in H_2_O: acetonitrile (90:10) (v/v) and B: 0.1% TFA in H_2_O: acetonitrile (10:90) (v/v). The elution gradient was 10%–90% B in 30 min. Fluorescence detection, excitation 345 and emission 530 nm.

For HPLC-FLd analysis, working solutions of curcumin were prepared by dilution with the mobile phase to final concentrations of 0.25, 0.5, 1.0, 2.0, 4.0, and 8.0 μg mL^−1^, all containing dansyl-l-phenylalanine at 5 μg mL^−1^, as an internal standard. The linearity of the detector responses for curcumin was determined for drug solutions and spiked tissues (liver), in duplicates, and the calibration curves were calculated by linear regression. The peak area ratio of curcumin to dansyl-l-phenylalanine was plotted against the concentration of curcumin.

*Biodistribution Studies of Curcumin in Major Organs*: Curcumin-loaded NCs were administered by a single intravenous injection at a dose of 16 mg kg^−1^ into CT26 tumor bearing Balb/c mice and were sacrificed after 1, 4, and 24 h (*n* = 3). Major organs (liver, lung, tumor, heart, kidney, and spleen) were removed and stored at −80 °C until curcumin was extracted as mentioned above for HPLC-FLd analysis. The amount of curcumin in the organs was determined in duplicates and a spiked tissue standard curve was included for each run. The data were expressed as the mean% ID/g of tissue ± SD.

*Tumor Growth Delay In Vivo Studies*: To determine the therapeutic action of NCs containing curcumin, tumor bearing Balb/c mice were randomly divided into four groups (*n* = 10) and anesthetized using isoflurane and injected *via* tail vein with (i) Saline; (ii) NC at 16 mg curcumin per kg in 200 μL; (iii) DOX at 5 mg kg^−1^ in 200 μL (positive control) or (iv) empty NC, all prepared in PBS and injected every 4 d for a total of four doses. Mice were sacrificed by cervical dislocation when tumors reached 1000 mm^3^. Data are given as mean value ± SEM, with *n* denoting the number of repeats. Significant differences were examined using one-way ANOVA. Tukey's multiple comparison test was further employed after one-way ANOVA for the tumor growth delay study. A *p* value of <0.05 was considered statistically significant in all studies.

*Histological Examination of Major Organs*: For histological examination of organs, Balb/c female mice were injected via tail vein with 200 μL of NCs (16 mg curcumin per kg) dispersed in PBS and tissues of tumor, kidney, liver, and spleen were excised when tumor volume reached 1000 mm^3^. Organs were immediately fixed in 10% neutral buffer formalin as 5 mm^2^ pieces. Two pieces of each organ were then paraffin-embedded and sectioned for haematoxylin and eosin stains (H&E) according to standard histological protocols at the Royal Veterinary College. All stained sections were analyzed using a Leica DM 1000 LED Microscope (Leica Microsystems, UK) coupled with CDD digital camera (Qimaging, UK).

*TUNEL Labeling Assay*: For detection of apoptosis, the DeadEnd Fluorometric TUNEL system was used to label nicked DNA through incorporation of fluorescein-12-dUTP. Samples were incubated with recombinant terminal deoxynucleotidyl transferase (rTdT) as per manufacturer's instructions, and fluorescein labeling was visualized using confocal laser mosaicing scanning microscopy (Nikon Eclipse Ti inverted microscope, Nikon, Tokyo, Japan) using an objective lens 20×/0.75NA 505-nm output filter, and a Plan-Neofluar 10 lens. DAPI VectaShield was used to counterstain nuclei. Each image consists of 256 × 256 pixels and the final mosaic consists of 12 × 15 images.

*Tumor Uptake Quantitative Analysis of Systemically Administered NC by Flow Cytometry*: For the quantification of curcumin uptake in tumor tissue, Balb/c female mice were injected via tail vein with 200 μL of NCs (16 mg curcumin per kg) dispersed in PBS. Tumor tissue processing was performed on ice and protected from light (whenever possible) in order to minimize the loss of viable cells and protect the fluorescent drug. Tumors were disrupted in small pieces using scissors and a scalpel and were homogenized by adding the pieces into Trypsin-EDTA 0.5% for 30 min at 37 °C (vortexed every 10 min) and passed through 10, 5, and 1 mL pipettes until pieces of tissue were no longer differentiated. Homogenates were further incubated in 5 mL of trypsin for 5 min at 37 °C. After, 20 mL of complete RPMI were added and tumor homogenates were then strained through 70 μm strainers (BD Biosciences), in order to obtain uniform cell suspensions. Cells were then transferred to 15 mL polystyrene tubes and washed twice with ice-cold PBS. From this cell suspension, 1 × 10^6^ cells were transferred to 5 mL polystyrene tubes and 50 000 cells were gated for the detection of curcumin using FL2 channel detector and BD FACS Calibur flow cytometer (BD Biosciences). The data were expressed as the mean ± SD.

*Statistical Analysis*: For all experiments, data were presented as mean ± SD except for therapy experiments; data were presented as mean ± SEM, where n denotes the number of repeats. Significant differences were examined using one-way ANOVA. *p* < 0.05 was considered statistically significant in all studies.

In case of therapy experiment, significant differences were examined using one-way ANOVA following by Tukey's multiple comparison test. * *p* < 0.05.

## Supporting Information

As a service to our authors and readers, this journal provides supporting information supplied by the authors. Such materials are peer reviewed and may be re-organized for online delivery, but are not copy-edited or typeset. Technical support issues arising from supporting information (other than missing files) should be addressed to the authors.

## References

[1] Shehzad A, Ul-Islam M, Wahid F, Lee YS (2014). J. Nanosci. Nanotechnol.

[2] Shieh YA, Yang SJ, Wei MF, Shieh MJ (2010). ACS Nano.

[3] Wang T, He N (2010). Nanoscale.

[4] Yurgel V, Collares T, Seixas F (2013). Braz. J. Med. Biol. Res.

[5] Moinard-Checot D, Chevalier Y, Briancon S, Fessi H, Guinebretiere S (2006). J. Nanosci. Nanotechnol.

[6] Lopez-Lazaro M (2008). Mol. Nutr. Food Res.

[7] Lin YG, Kunnumakkara AB, Nair A, Merritt WM, Han LY, Armaiz-Pena GN, Kamat AA, Spannuth WA, Gershenson DM, Lutgendorf SK, Aggarwal BB, Sood AK (2007). Clin. Cancer Res.

[8] Dorai T, Cao YC, Dorai B, Buttyan R, Katz AE (2001). Prostate.

[9] Aggarwal S, Ichikawa H, Takada Y, Sandur SK, Shishodia S, Aggarwal BB (2006). Mol. Pharmacol.

[10] Menon LG, Kuttan R, Kuttan G (1999). Cancer Lett.

[11] Anand P, Kunnumakkara AB, Newman RA, Aggarwal BB (2007). Mol. Pharm.

[12] Sharma RA, Euden SA, Platton SL, Cooke DN, Shafayat A, Hewitt HR, Marczylo TH, Morgan B, Hemingway D, Plummer SM, Pirmohamed M, Gescher AJ, Steward WP (2004). Clin. Cancer Res.

[13] Yallapu MM, Gupta BK, Jaggi M, Chauhan SC (2010). J. Colloid Interface Sci.

[14] Ghalandarlaki N, Alizadeh AM, Ashkani-Esfahani S (2014). Biomed. Res. Int.

[15] Li L, Xiang D, Shigdar S, Yang W, Li Q, Lin J, Liu K, Duan W (2014). Int. J. Nanomed.

[16] Ivanov PI, Bontchev GD, Bozhikov GA, Filossofov DV, Maslov OD, Milanov MV, Dmitriev SN (2003). Appl. Radiat. Isot.

[17] Jayaprakasha GK, Jagan MohanLRao, Sakariah KK (2002). J. Agri. Food Chem.

[18] Kunnumakkara AB, Guha S, Krishnan S, Diagaradjane P, Gelovani J, Aggarwal BB (2007). Cancer Res.

[19] Dhillon N, Aggarwal BB, Newman RA, Wolff RA, Kunnumakkara AB, Abbruzzese JL, Ng CS, Badmaev V, Kurzrock R (2008). Clin. Cancer Res.

[20] Kunnumakkara AB, Anand P, Aggarwal BB (2008). Cancer. Lett.

[21] Bouchemal K, Briancon S, Perrier E, Fessi H (2004). Int. J. Pharm.

[22] Mosqueira VC, Legrand P, Pinto-Alphandary H, Puisieux F, Barratt G (2000). J.Pharm. Sci.

[23] Fasina OO, Colley Z (2008). Int. J. Food Prop.

[24] Neelamegam P, Krishnaraj S (2011). Indian J. Chem. Technol.

[25] Sa G, Das T (2008). Cell Div.

[26] Karunagaran D, Rashmi R, Kumar TR (2005). Curr Cancer Drug Targets.

[27] Basnet P, Skalko-Basnet N (2011). Molecules.

[28] Yallapu MM, Jaggi M, Chauhan SC (2012). Drug Discovery Today.

[29] Bisht S, Feldmann G, Soni S, Ravi R, Karikar C, Maitra A, Maitra A (2007). J. Nanobiotechnol.

[30] Sahu A, Bora U, Kasoju N, Goswami P (2008). Acta Biomater.

[31] Verderio P, Bonetti P, Colombo M, Pandolfi L, Prosperi D (2013). Biomacromolecules.

[32] Mukerjee A, Vishwanatha JK (2009). Anticancer Res.

[33] Prajakta D, Ratnesh J, Chandan K, Suresh S, Grace S, Meera V, Vandana P (2009). J. Biomed. Nanotechnol.

[34] Baskaran R, Madheswaran T, Sundaramoorthy P, Kim HM, Yoo BK (2014). Int. J. Nanomed.

[35] Anitha A, Sreeranganathan M, Chennazhi KP, Lakshmanan VK, Jayakumar R (2014). Eur. J. Pharm. and Biopharm.

[36] Chuah LH, Roberts CJ, Billa N, Abdullah S, Rosli R (2014). Colloids Surf B: Biointerfaces.

[37] Jiang Z, Jin S, Yalowich JC, Brown KD, Rajasekaran B (2010). Mol. Cancer Therapy.

[38] Thomas CP, Pillai LS, Krishnan L (2014). J. Cancer Therapy.

[39] Mulik RS, Monkkonen J, Juvonen RO, Mahadik KR, Paradkar AR (2012). Int. J. Pharm.

[40] Mirgani TM, Isacchi B, Sadeghizadeh M, Marra F, Bilia AR, Mowla SJ, Najafi F, Babaei E (2014). Int. J. Nanomed.

[41] Jambhrunkar S, Qu Z, Popat A, Yang J, Noonan O, Acauan L, Ahmad YNor, Yu C, Karmakar S (2014). Mol. Pharm.

[42] Mehta K, Pantazis P, McQueen T, Aggarwal BB (1997). Anticancer Drugs.

[43] Choudhuri T, Pal S, Das T, Sa G (2005). J. Biol. Chem.

[44] Moragoda L, Jaszewski R, Majumdar AP (2001). Anticancer Res.

[45] Kumar SS, Mahesh A, Mahadevan S, Mandal AB (2014). Biochim. Biophys. Acta.

[46] Misra R, Sahoo SK (2011). Mol. Pharm.

[47] Yallapu MM, Khan S, Maher DM, Ebeling MC, Sundram V, Chauhan N, Ganju A, Balakrishna S, Gupta BK, Zafar N, Jaggi M, Chauhan SC (2014). Biomaterials.

[48] Yallapu MM, Maher DM, Sundram V, Bell MC, Jaggi M, Chauhan SC (2010). J. Ovarian Res.

[49] Yin HT, Zhang DG, Wu XL, Huang XE, Chen G (2013). Asian Pacific Journal of Cancer Prevention: APJCP.

[50] Duan J, Zhang Y, Han S, Chen Y, Li B, Liao M, Chen W, Deng X, Zhao J, Huang B (2010). Int. J. Pharm.

[51] Mazzarino L, Silva LF, Curta JC, Licinio MA, Costa A, Pacheco LK, Siqueira JM, Montanari J, Romero E, Assreuy J, Santos-Silva MC, Lemos-Senna E (2011). J. Biomed. Nanotechnol.

[52] Zanotto-Filho A, Coradini K, Braganhol E, Schroder R, de Oliveira CM, Simoes-Pires A, Battastini AM, Pohlmann AR, Guterres SS, Forcelini CM, Beck RC, Moreira JC (2013). Eur. J. Pharm. Biopharm.

[53] Khalil NM, do Nascimento TC, Casa DM, Dalmolin LF, de Mattos AC, Hoss I, Romano MA, Mainardes RM (2013). Colloids Surf. B: Biointerfaces.

[54] Anand P, Nair HB, Sung B, Kunnumakkara AB, Yadav VR, Tekmal RR, Aggarwal BB (2010). Biochem. Pharmacol.

[55] Beloqui A, Coco R, Memvanga PB, Ucakar B, des Rieux A, Preat V (2014). International J. Pharm.

[56] Mathew A, Fukuda T, Nagaoka Y, Hasumura T, Morimoto H, Yoshida Y, Maekawa T, Venugopal K, Kumar DS (2012). PloS One.

[57] Shaikh J, Ankola DD, Beniwal V, Singh D, Kumar MN (2009). Eur. J. Pharm. Sci.

[58] Ranjan AP, Mukerjee A, Helson L, Vishwanatha JK (2012). J. Nanobiotechnol.

[59] Punfa W, Suzuki S, Pitchakarn P, Yodkeeree S, Naiki T, Takahashi S, Limtrakul P (2014). Asian Pacific J. Cancer Prev.

[60] Lu WD, Qin Y, Yang C, Li L, Fu ZX (2013). Clinics.

[61] Tonnesen HH, Masson M, Loftsson T (2002). Int. J. Pharm.

[62] Li L, Braiteh FS, Kurzrock R (2005). Cancer.

[63] Apiratikul N, Penglong T, Suksen K, Svasti S, Chairoungdua A, Yingyongnarongkula B (2013). Bioorganicheskaia khimiia.

[64] Bisht S, Mizuma M, Feldmann G, Ottenhof NA, Hong SM, Pramanik D, Chenna V, Karikari C, Sharma R, Goggins MG, Rudek MA, Ravi R, Maitra A, Maitra A (2010). Mol. Cancer Ther.

[65] Mulik RS, Monkkonen J, Juvonen RO, Mahadik KR, Paradkar AR (2010). Mol. Pharm.

[66] Gou M, Men K, Shi H, Xiang M, Zhang J, Song J, Long J, Wan Y, Luo F, Zhao X, Qian Z (2011). Nanoscale.

[67] Frank LA, Contri RV, Beck RC, Pohlmann AR, Guterres SS (2015). WIREs Nanomed Nanobiotechnol.

[68] Sharma NBM, Visht S, Sharma PK, Kulkarni GT (2010). Chron. Young Sci.

[69] Maestrelli F, Mura P, Alonso MJ (2004). J. Microencapsul.

[70] Mastrobattista E, Koning GA, Storm G (1999). Adv. Drug Delivery Rev.

[71] Ravichandran R (2013). J. Biomater. Nanobiotechnol.

[72] Moghimi SM, Hunter AC, Murray JC (2001). Pharmacol. Rev.

[73] Tsai YM, Chien CF, Lin LC, Tsai TH (2011). Int. J.l Pharm.

[74] Patra D, Barakat C (2011). Spectrochim. Acta, Part A: Mol. Biomol. Spectrosc.

[75] Fessi HPF, Devissaguet J, Ammoury N, Benita S (1989). Int. J. Pharm.

[76] Anuchapreeda S, Fukumori Y, Okonogi S, Ichikawa H (2011). J. Nanotechnol.

[77] Jasanada F, Urizzi P, Souchard JP, Le FGaillard, Favre G, Nepveu F (1996). Bioconjugate Chem.

[78] Al-Jamal KT, Nunes A, Methven L, Ali-Boucetta H, Li S, Toma FM, Herrero MA, Al-Jamal WT, ten Eikelder HM, Foster J, Mather S, Prato M, Bianco A, Kostarelos K (2012). Angew. Chem. Int. Ed. Engl.

