# Supplementary material for: Passively Targeted Curcumin-Loaded PEGylated PLGA Nanocapsules for Colon Cancer Therapy In Vivo
Source: Small. 2015 Jul 3;11(36):4704–22. doi: 10.1002/smll.201403799 (PMC4660879; doi:10.1002/smll.201403799)
Supplement: Supplementary file 1 — Supplementary [file smll0011-4704-sd1.pdf]

Copyright WILEY-VCH Verlag GmbH & Co. KGaA, 69469 Weinheim, Germany, 2015.

NANO small MICRO

Supporting Information

for *Small.,* DOI: 10.1002/smll.201403799

Passively Targeted Curcumin-Loaded PEGylated PLGA Nanocapsules for Colon Cancer Therapy In Vivo

*Rebecca Klippstein, Julie Tzu-Wen Wang, Riham I. El-Gogary, Jie Bai, Falisa Mustafa, Noelia Rubio, Sukhvinder Bansal, Wafa T. Al-Jamal, and Khuloud T. Al-Jamal**

DOI: 10.1002/ ((please add manuscript number))
Full Paper

Passively Targeted Curcumin-Loaded PEGylated PLGA Nanocapsules for Colon Cancer Therapy *In Vivo*

*Rebecca Klippstein, Julie Tzu-Wen Wang, Riham I. El-Gogary, Jie Bai, Falisa Mustafa, Noelia Rubio, Sukhvinder Bansal, Wafa T. Al-Jamal and Khuloud T. Al-Jamal**

Dr. R. Klippstein, Dr. J. Wang, J. Bai, F. Mustafa, Dr. N. Rubio-Carrero, Dr. S. Bansal, Dr. K.T. Al-Jamal
150 Stamford Street,
Institute of Pharmaceutical Science
King's College London
Franklin-Wilkins Building
150 Stamford Street
London SE1 9NH, UK
E-mail: khuloud.al-jamal@kcl.ac.uk

Dr. Riham I. El-Gogary
Ain Shams University
Department of Pharmaceutics and Industrial Pharmacy Faculty of Pharmacy
Ain Shams University
Khalifa El-Maamon Street
Abbasiya Square, Cairo 11566, Egypt

Dr. W.T. Al-Jamal
School of Pharmacy,
University of East Anglia, Norwich Research Park,
Norwich NR4 7TJ, UK

Supplementary Information

Table S1: Physico-chemical characterization of DTPA-PLGA NCs.

Formulation	Composition	Hydrodynamic diameter (nm)[a,b]	PDI ±SD [a,b]	Zeta potential ±SD (mV) [b]
**NC (non-PEGylated)**	PLGA-COOH (100%)	153.0 ± 4.30	0.11 ± 0.01	-46.2 ± 1.50
**DTPA-NC (non-PEGylated)**	PLGA-COOH:PLGA-NH-PEG-NH-DTPA (95%: 5%)	152.5 ± 0.99	0.11± 0.02	-44.2 ± 2.07
**NC**	PLGA-NH-PEG-NH_2 (100%)	143.1 ± 1.71	0.13 ±0.01	-32.9 ± 1.82
**DTPA-NC**	PLGA-NH-PEG-NH_2:PLGA-NH-PEG-NH-DTPA (95%: 5%)	142.2 ± 1.14	0.13 ± 0.01	-35.6 ± 0.49

[a] Measured by dynamic light scattering.
[b] Expressed as mean ± SD (n=3).

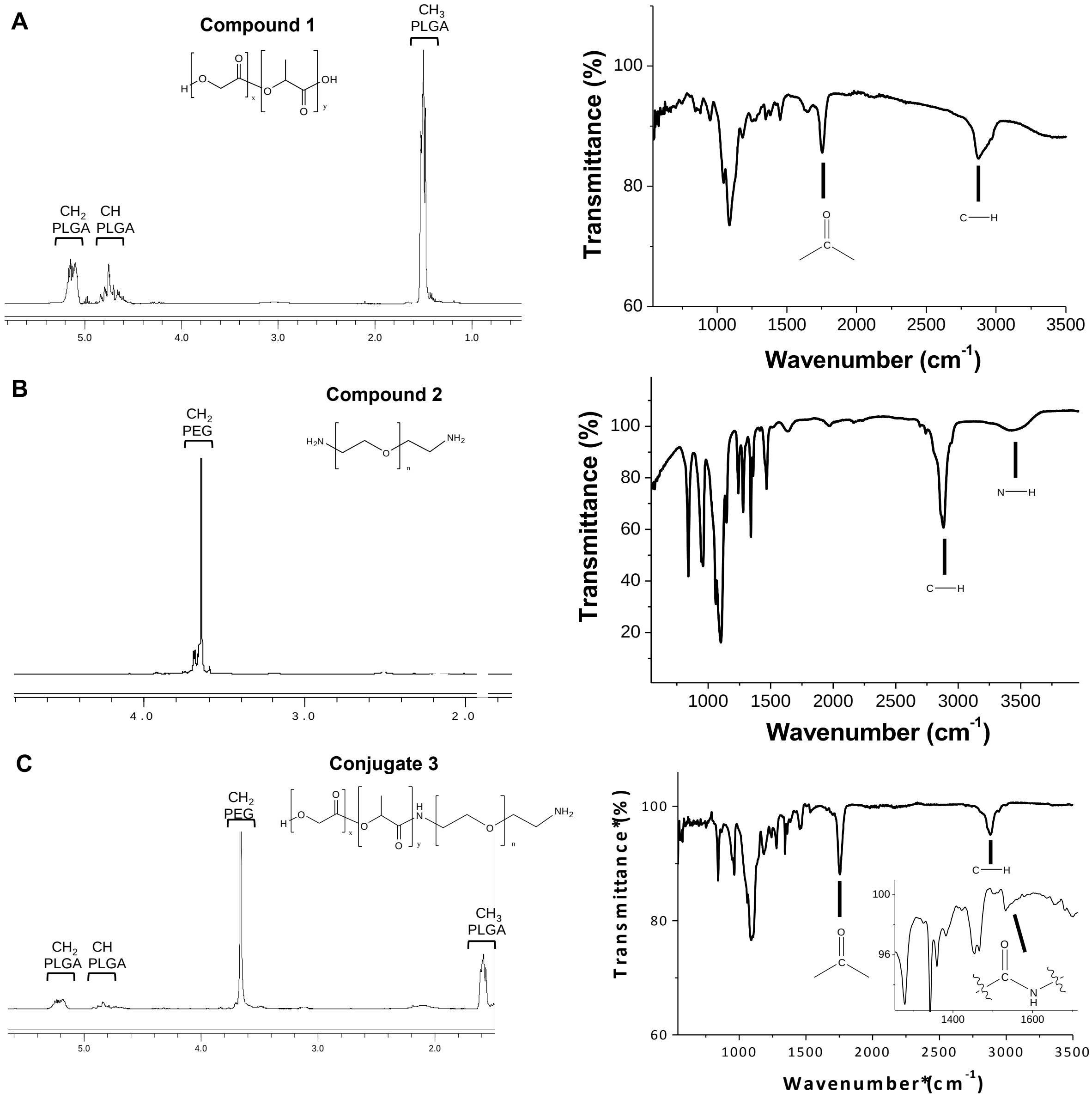

Figure S1. Characterization of the conjugates with ^{1}H-NMR and FT-IR. ^{1}H-NMR spectra ($CDCl_3$) and FT-IR spectra (ATR mode) of **(A)** compound **1** ($PLGA_{18KDa}$), **(B)** compound **2** (NH_2-$PEG_{3.5KDa}$-NH_2) and **(C)** conjugates **3** ($PLGA_{18KDa}$-$PEG_{3.5KDa}$-NH_2).

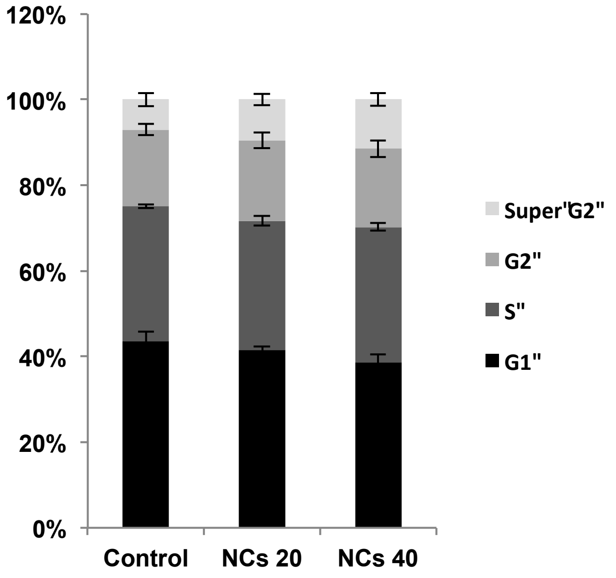

Figure S2. CT26 murine colon cancer cell cycle distribution analysis. CT26 cells were incubated with NC for 24 h at 20 and 40 μM drug concentration. Relative changes in the percentage of cell cycle phases following 24 h of drug treatment are presented. No significant changes in cell cycle phase distribution were found. Values are expressed as mean ± SD (n=3).

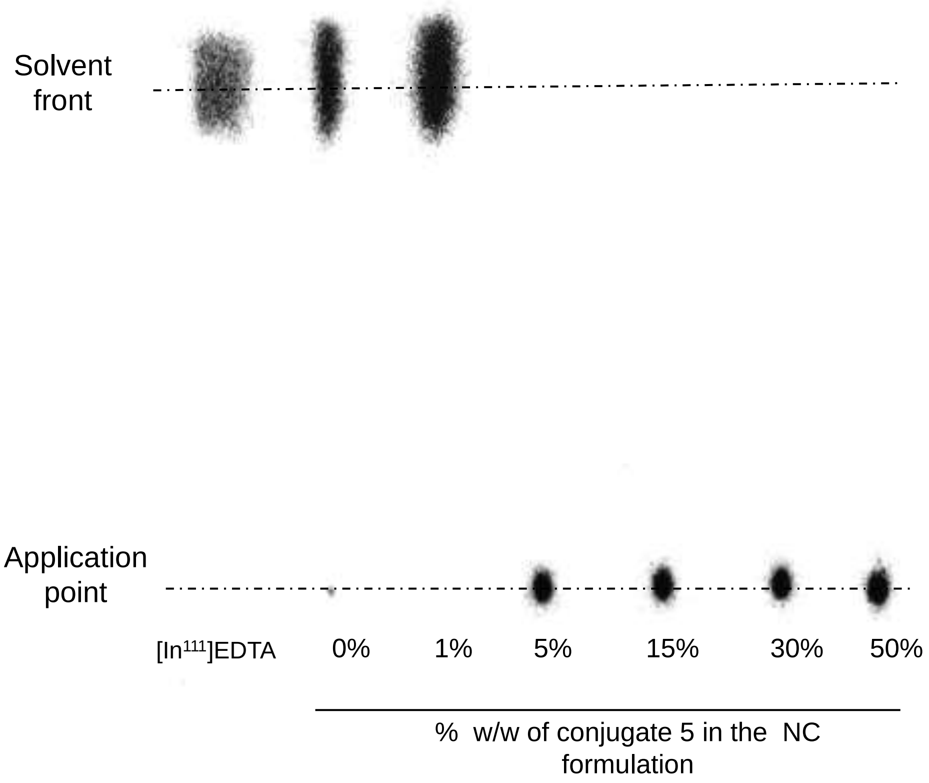

Figure S3: Optimisation of radio-labelling of [^{111}In]-labelled NC. NCs were prepared by increasing the contents of conjugate 5 in the formulation in order to optimise the radio-labelling efficiency while not affecting the NC's size and charge.

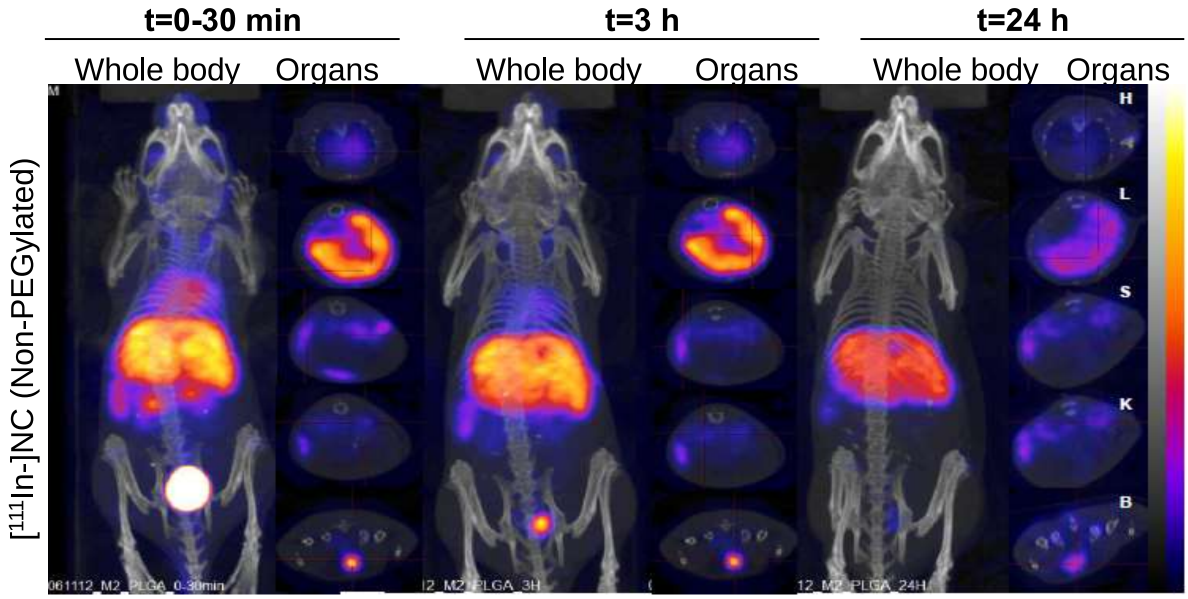

Figure S4. ***In vivo*** **whole body 3D SPECT/CT imaging of NC-^{111}In in CT26 tumor-bearing balb/c.** Mice were i.v injected with NC-^{111}In (non-PEGylated) at a dose of 610 mg polymer/Kg. Mice for SPECT/CT imaging with tumor inoculated at one side only while tumor inoculated bifocally for gamma scintigraphy studies. Whole body 3D SPECT/CT imaging at 0-30min, 4 and 24 h post-injection with scanning time of 40-60 min each. Cross-sections were from heart (H), liver (L), spleen (S), kidney (KI) and tumor (T) at equivalent time points. Tumor accumulation was observed at 4 h post injection and was enhanced over time.

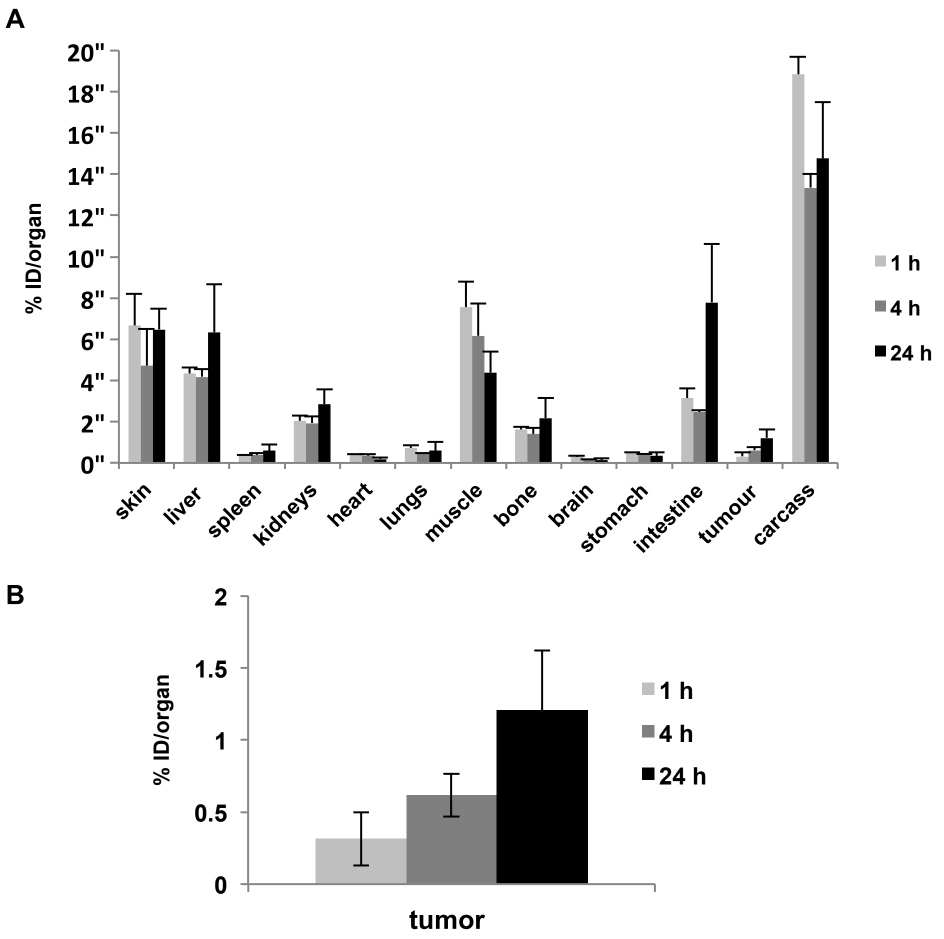

Figure S5: *In vivo* biodistribution of the radio-labelled NC in CT26 tumor bearing Balb/C mice after single dose administration *via* tail vein. (A) Results were expressed as percentage injected dose per organ (%ID/organ) at 1, 4 and 24 h after injection of 600 mg polymer/Kg. **(B)** The uptake in tumors. Data were expressed as means ± SD (*n*=3-4).

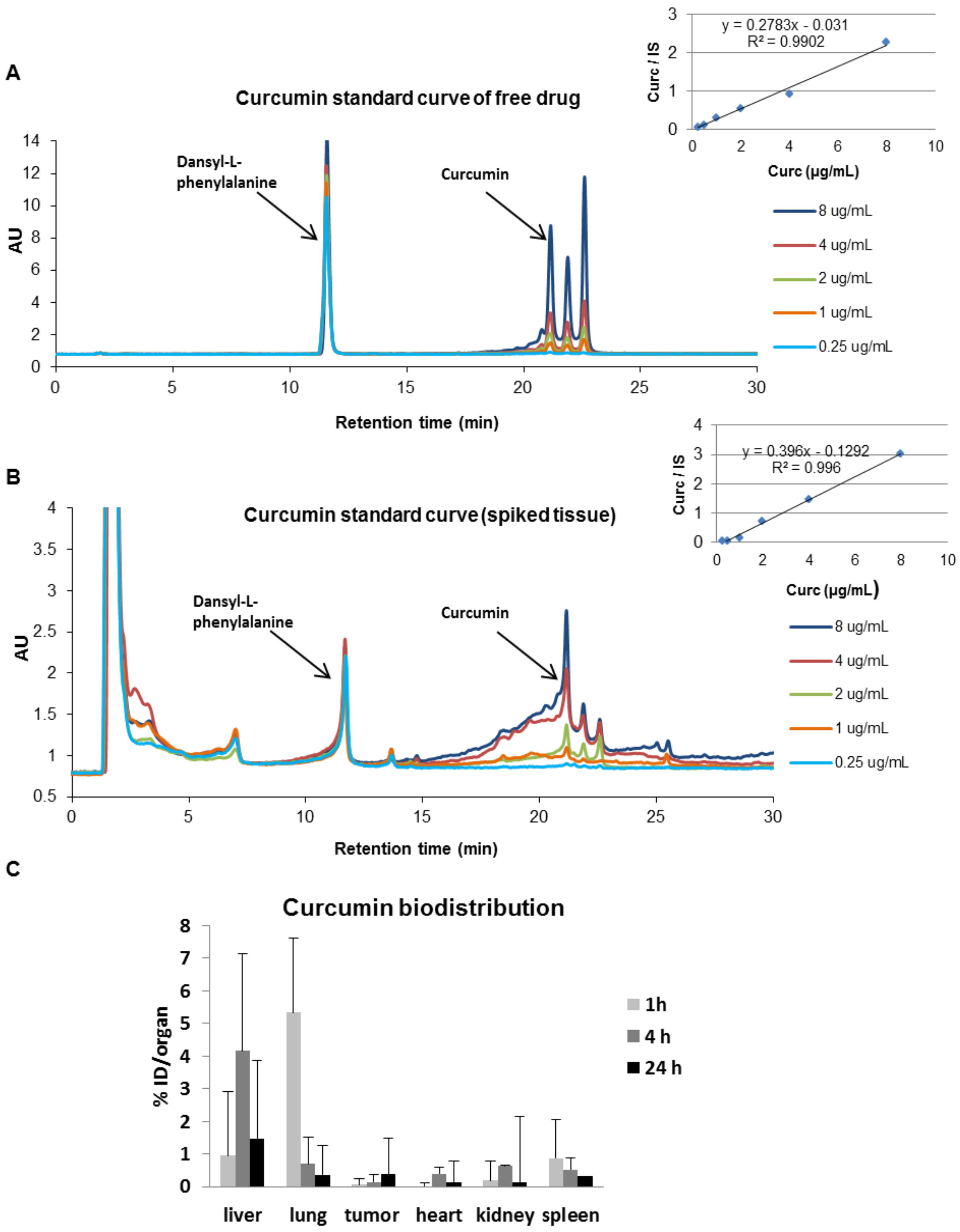

Figure S6. HPLC-FLd chromatograms of dansyl-L-phenylalanine and curcumin and biodistribution of curcumin in major organs. HPLC chromatograms and linear regression plots of different concentrations of curcumin (from 0.25 to 8 µg/mL) at a fixed concentration of dansyl-L-phenylalanine (5 µg/mL) prepared from **(A)** free drug or **(B)** drug spiked liver samples. Curcumin, demetoxycurcumin and di-demethoxycurcumin peaks are observed at retention times of 21.2, 21.8 and 22.5 min. **(C)** *In vivo* biodistribution of curcumin-loaded NCs after a single dose administration *via* tail vein of CT-26 tumor-bearing mice. Curcumin

was extracted from major organs (liver, lung, tumor, heart, kidneys and spleen) at 1, 4 and 24 h post-injection. Major organ biodistribution profile values were expressed as percentage injected dose per organ (%ID/organ). Results are expressed as means ± SD (n=3).
